# Secondary injury and inflammation after intracerebral haemorrhage: a systematic review and meta-analysis of molecular markers in patient brain tissue

**DOI:** 10.1136/jnnp-2021-327098

**Published:** 2021-08-06

**Authors:** James JM Loan, Caoimhe Kirby, Katherine Emelianova, Owen R Dando, Michael TC Poon, Leisan Pimenova, Giles E Hardingham, Barry W McColl, Catharina JM Klijn, Rustam Al-Shahi Salman, Floris HBM Schreuder, Neshika Samarasekera

**Affiliations:** 1 Centre for Clinical Brain Sciences, The University of Edinburgh, Edinburgh, UK; 2 Centre for Discovery Brain Sciences, The University of Edinburgh, Edinburgh, UK; 3 UK Dementia Research Institute at Edinburgh, The University of Edinburgh, Edinburgh, UK; 4 The Usher Institute, The University of Edinburgh, Edinburgh, UK; 5 Neurosurgery, München Klinik, gGmbH, Munich, Germany; 6 Department of Neurology, Donders Institute for Brain, Cognition and Behaviour, Radboud University Medical Centre, Nijmegen, Netherlands

**Keywords:** meta-analysis, stroke, systematic reviews, neuropathology, immunology

## Abstract

**Background:**

Inflammatory responses to intracerebral haemorrhage (ICH) are potential therapeutic targets. We aimed to quantify molecular markers of inflammation in human brain tissue after ICH compared with controls using meta-analysis.

**Methods:**

We searched OVID MEDLINE (1946–) and Embase (1974–) in June 2020 for studies that reported any measure of a molecular marker of inflammation in brain tissue from five or more adults after ICH. We assessed risk of bias using a modified Newcastle-Ottawa Scale (mNOS; mNOS score 0–9; 9 indicates low bias), extracted aggregate data, and used random effects meta-analysis to pool associations of molecules where more than two independent case–control studies reported the same outcome and Gene Ontology enrichment analysis to identify over-represented biological processes in pooled sets of differentially expressed molecules (International Prospective Register of Systematic Reviews ID: CRD42018110204).

**Results:**

Of 7501 studies identified, 44 were included: 6 were case series and 38 were case–control studies (median mNOS score 4, IQR 3–5). We extracted data from 21 491 analyses of 20 951 molecules reported by 38 case–control studies. Only one molecule (interleukin-1β protein) was quantified in three case–control studies (127 ICH cases vs 41 ICH-free controls), which found increased abundance of interleukin-1β protein after ICH (corrected standardised mean difference 1.74, 95% CI 0.28 to 3.21, p=0.036, I^2^=46%). Processes associated with interleukin-1β signalling were enriched in sets of molecules that were more abundant after ICH.

**Conclusion:**

Interleukin-1β abundance is increased after ICH, but analyses of other inflammatory molecules after ICH lack replication. Interleukin-1β pathway modulators may optimise inflammatory responses to ICH and merit testing in clinical trials.

## Introduction

Stroke due to spontaneous intracerebral haemorrhage (ICH) causes substantial death and disability.^1^ There are no effective specific medical treatments for ICH.^2^

Brain inflammation is a potential therapeutic target after ICH. Much of our understanding of the inflammatory response in the brain after ICH has been informed by studies of animal models.^3^ In rodent models, modulators of inflammatory responses to ICH can be used to improve functional outcome.^3^ Although animal models provide useful mechanistic insights, there are significant differences in immune physiology, haemorrhage induction and haemorrhage evolution between humans and rodents with ICH.^4 5^ To aid in the design and selection of interventions for clinical trials that translate the benefits of interventions tested in experimental ICH into humans, greater knowledge of disease processes in humans after ICH is needed.

To identify potential therapeutic targets for ICH, we conducted a systematic review and meta-analysis of inflammation in human brain tissue following ICH with three aims: first, to identify all molecular measures of inflammation reported by published studies of ICH in human brain tissue; second, to quantify pooled associations between individual molecules and ICH compared with controls; and third, to identify associations between ICH and groups of molecules with similar functions, compared with controls.

## Methods

### Design

We performed a systematic review and meta-analysis. Our study protocol was prospectively registered with the International Prospective Register of Systematic Reviews (CRD42018110204).

### Search strategy and selection criteria

**Figure 1:**
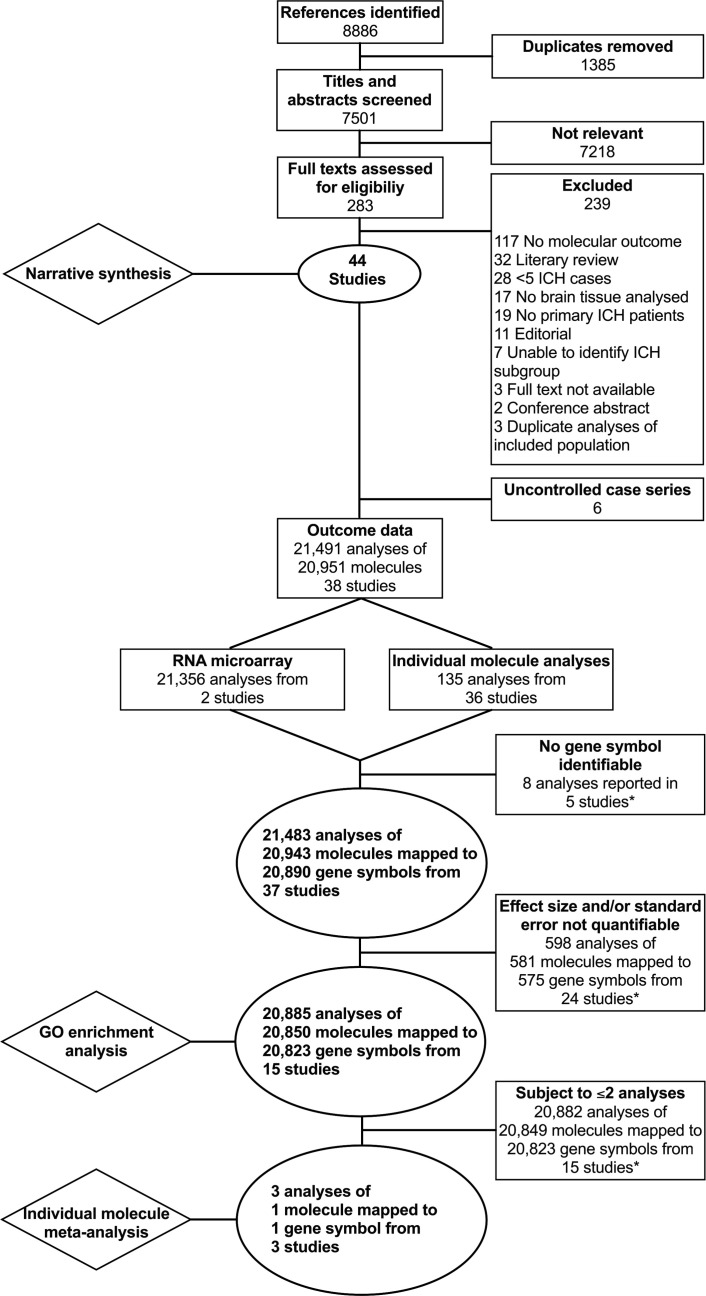
Flow diagram of the study selection. *Exclusion of ineligible analyses only. Other eligible analyses from the same study remain included. GO, Gene Ontology; ICH, intracerebral haemorrhage.

JJML searched Ovid MEDLINE (1946) and Ovid Embase (1974) on 18 June 2020 using terms to identify studies of molecular measures of inflammation in brain tissue from human patients with ICH (online supplemental table 1). We did not apply any language limits to searches and did not use hand searching or searches of grey literature. We extracted all records yielded by these searches to Covidence, where duplicates were removed.^6^ JJML and CK independently screened the titles and abstracts to identify reports of potentially eligible studies for full-text review. We included cohort studies, case–control studies and case series which reported at least one summary molecular measure of inflammation in adult (age ≥16 years) human brain tissue after spontaneous ICH in any language. JJML reviewed the bibliographies of each included study for potentially eligible study reports and searched for potentially eligible forward citations using Google Scholar (scholar.google.com). Translators reviewed titles and abstracts before being interviewed by JJML and CK, who made decisions regarding eligibility. We excluded studies of ICH due to an underlying macrovascular cause, trauma or hereditary cerebral amyloid angiopathy (CAA); studies reporting mixed aetiologies of haemorrhage where spontaneous ICH could not be distinguished from other causes; studies of fewer than five cases; conference abstracts; and studies which did not report a molecular measure of brain inflammation (figure 1). We contacted the corresponding authors of studies for which it was unclear if relevant data had been yielded and included these if it was forthcoming. At each screening stage, translators were recruited to translate into English any titles, abstracts and full texts published in other languages (acknowledgements and contributions). A third independent reviewer (FHBMS) made the final decision over inclusion where there were conflicts at the title, abstract and full-text screening stages.

10.1136/jnnp-2021-327098.supp1 Supplementary data

### Data extraction and analysis

JJML and CK independently extracted data from included full texts using standardised proformas (online supplemental table 2). Summary measures of expression of molecules were extracted for ICH cases, control brain tissue from ICH cases that was distant from the haematoma and control brain tissue from non-ICH controls. Data derived from any analytical technique using any molecule were extracted with the exception of analyses of amyloid-beta protein, which we did not consider a marker of neuroinflammatory response to ICH as its accumulation is known to often precede ICH onset.^7^ JJML and CK independently assessed the risk of bias of included case–control studies using a modified Newcastle-Ottawa Quality Assessment Scale for case–control studies (Newcastle-Ottawa Scale (mNOS), online supplemental table 3).^8^ This assessment assigned points for factors indicating low risk of bias, and scores ranged from 0 to 9. We modified the Newcastle-Ottawa Quality Assessment Scale for use in pathological studies of ICH by introducing three additional point scoring criteria: one for use of control tissue distant to ICH from ICH cases, another for efforts to eliminate or control for potential batch effects in tissue processing and a final one for complete reporting of all cases and control data. We used a single item to assess case definition and ascertainment of exposure. Non-response rate was not assessed. We summarised the characteristics and extracted summary neuroinflammatory outcome measures from all included studies in a narrative synthesis.

We undertook a meta-analysis of associations of individual molecules with ICH where more than two studies had analysed the same molecule and reported numerical measures of association or expression as well as SE for ICH and control tissue. We used the R package ‘Meta’ V.15–1 in RStudio V.1.3.1093 running R Core V.3.6.1 to undertake random-effects meta-analysis of continuous outcome data and produce forest and funnel plots. For random-effects meta-analysis, we calculated variance of true effects, *τ* ^2^, using the empirical Bayes estimator^9^ and used the Hartung and Knapp corrected standardised mean difference as the main summary outcome measure.^10^ We used the I^2^ statistic to estimate between-studies heterogeneity.

We next attempted to link all molecules analysed by all included studies to Human Gene Organisation Gene Nomenclature Committee (HGNC)-approved gene symbols. For this, we searched Laboratory of Human Retrovirology and Immunoinformatics Database for Annotation, Visualisation and Integrated Discovery Gene ID conversion tool V.6.8, the HGNC multisymbol checker, the US National Centre for Biotechnology Information Gene list and AceView for molecules described by included studies.^11–14^ We excluded non-specific analyses of multimeric nuclear factor kappa B (NF-κB) and human leukocyte antigen (HLA) protein and analyses of lipids which could not be linked to a single gene symbol as well as uncontrolled analyses (figure 1). We classified associations between gene symbols identified in each study as positive if the fold change in expression between ICH and control tissue was greater than 1.5, negative if the fold change was less than 2/3 and neutral if it was equal to or between 2/3 and 1.5.^15^ Where numerical data could not be extracted from studies, we used reported statistical significance to characterise associations as positive, negative or neutral. If multiple control groups were described, results from control brain tissue from the same case as the ICH were used in preference to control tissue derived from unaffected individuals. We selected peak or trough fold changes where multiple time points were described. If duplicate analyses of overlapping populations were described by multiple study reports, we selected the largest population per analysis.

We tested for Gene Ontology (GO) biological process term enrichment using the package topGO V.2.41.0. GO enrichment analysis was performed for subsets of genes that had positive or negative associations with ICH, partitioned according to the number of studies repeating analysis of the same gene symbol showing the same direction of association. Gene symbols derived from studies that were at high risk of bias (mNOS score 0–3) were excluded from this analysis. This analysis used topGO’s ‘weight01’ algorithm, a mixture of the ‘elim’ and ‘weight’ algorithms which aims to increase the specificity of GO terms identified and to reduce bias arising from dependence of neighbouring GO terms on each other. The enrichment score numerator for each GO term is defined as the number of genes found in a meta-analysis set, and the denominator is the total number of genes annotated for each GO term in the background gene set. Statistical significance of normalised enrichment is determined using Fisher’s exact test. The background gene set was defined as genes detected at a mean expression of more than one fragment per kilobase million mapped reads by RNA sequencing of seven human brain samples obtained at postmortem following the sudden death of patients due to non-neurological disease (online supplemental table 4). These patients were identified retrospectively from the Sudden Death Brain and Tissue Banks that are part of Medical Research Council Edinburgh and Manchester Brain and Tissue Banks. All patients underwent postmortem examination of brain tissue to exclude acute neurological disease as a cause of death. Cases with frozen tissue available with a sample RNA integrity number >5 were selected on the basis of similar age range (median 72 years, IQR 71.0–74.5) and coexisting small vessel diseases to those associated with ICH (mild–moderate non-amyloid arteriosclerosis, n=4; mild spontaneous CAA, n=1; no abnormality, n=3).^7 16 17^ RNA was extracted and purified from whole brain tissue homogenate using the RNeasy Lipid Tissue Mini kit (Qiagen, 74804). TruSeq stranded messenger RNA (mRNA)-seq libraries were created per sample (Illumina), which were then each sequenced to a depth of 50 million 50 bp paired-end reads (NovaSeq 6000, Illumina). Read alignment with the human genome was then performed as previously described.^18 19^ Chord diagrams were produced using the R Circlize package V.0.4.11.

## Results

**Figure 2:**
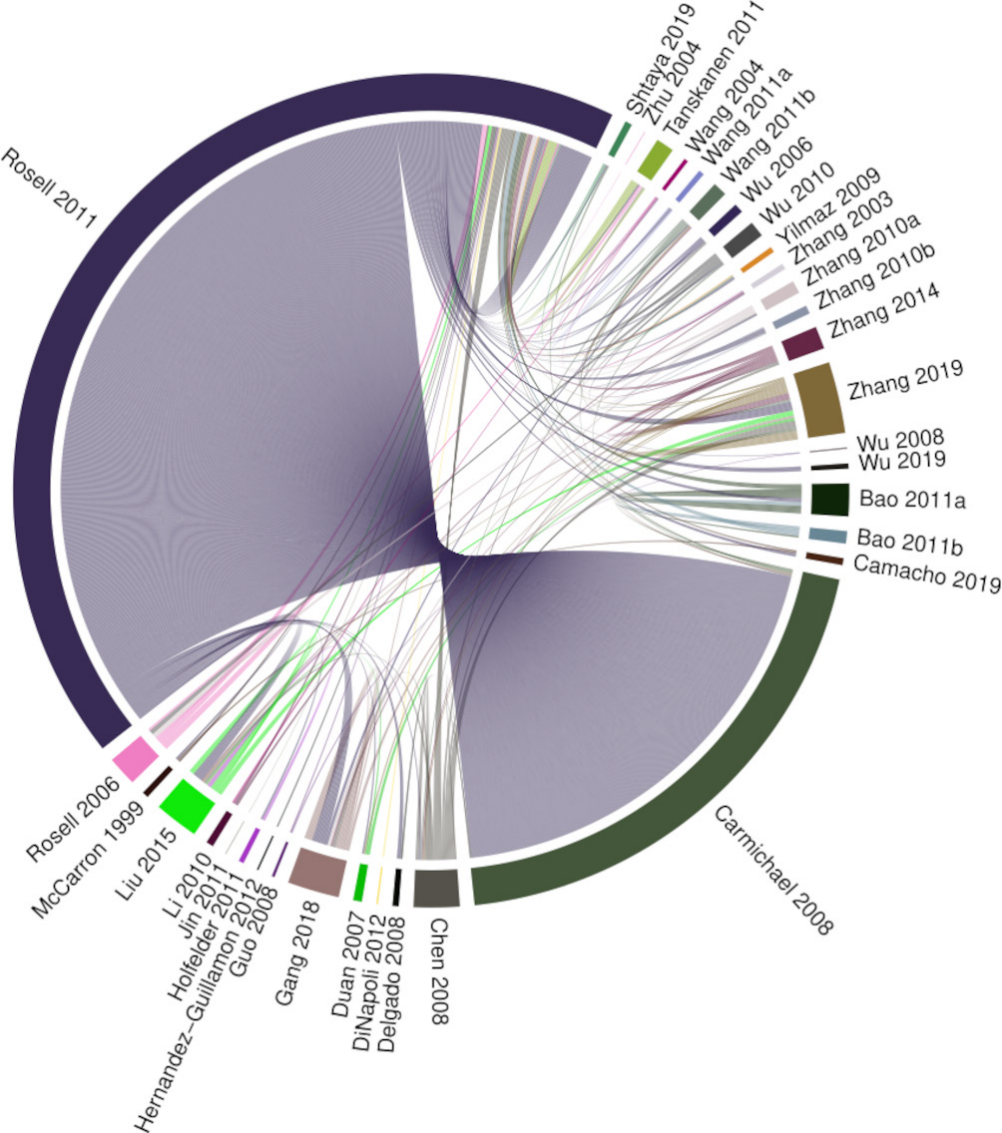
Chord diagram of replicated findings. This demonstrates which studies conducted analyses of the same gene symbols and indicates that many replicated analyses were derived from two studies.^20 21^ Segments indicate studies reporting analyses of a gene symbol that has been analysed more than once. Chords link segments reporting the same molecule. Chord width is weighted by risk of bias, with larger width indicating lower risk of bias.

Our searches identified 8886 records, of which 7501 were unique. Of these records, 7218 were excluded after a review of titles and abstracts. Of the 283 full texts reviewed, 239 were excluded (figure 1). In total, we identified 44 eligible study reports for inclusion in our narrative synthesis (online supplemental tables 5 and 6). Six uncontrolled case series were included in the narrative review but were excluded from further analysis. The median mNOS score in the remaining 38 case–control studies was 4 (IQR 3–5, online supplemental table 7). These studies reported the outcomes of 21 491 analyses of 20 951 molecules. A total of 21 356 analyses were derived from two studies^20 21^ which used microarray to measure RNA transcript abundance, and 135 were from the remaining 36 studies. Full data from one microarray study were available from an online repository.^20^ However, only published data concerning significantly differentially associated microarray probes were available for the other microarray study, which did not include measures of SE.^21^ The corresponding author was unable to provide additional data. Eight analyses conducted by five studies could not be assigned a gene symbol. Gene symbols were identified for the remaining 21 483 analyses of protein and RNA by 37 studies. In this dataset, 539 gene symbols (1134 analyses, in 35 studies) were subject to more than one analysis (figure 2). Numerical measures of effect size and/or SE were not available for 598 analyses reported by 24 studies and so could not be used for meta-analysis. The final dataset for meta-analysis comprised 20 885 analyses of 20 850 molecules mapped to 20 823 gene symbols and reported by 15 studies (online supplemental table 7).

### Individual molecule meta-analysis

Just one molecule met criteria for meta-analysis. Interleukin-1β (IL-1β) protein was analysed by three independent studies.^22–24^ These studies analysed IL-1β abundance in brain tissue obtained during surgical treatment of ICH using 3,3′-diaminobenzidine immunohistochemistry (IHC). As a control sample, two studies used surgically resected brain tissue obtained during surgery for non-haemorrhagic, non-malignant disease,^22 24^ and the other used tissue that was obtained during ICH surgery that had a margin of at least 1 cm from the haematoma border.^23^ We scored two of these studies at 4^23 24^ points and the other at 5^22^ out of a maximum of 9 points in our mNOS risk of bias assessment.

**Figure 3:**
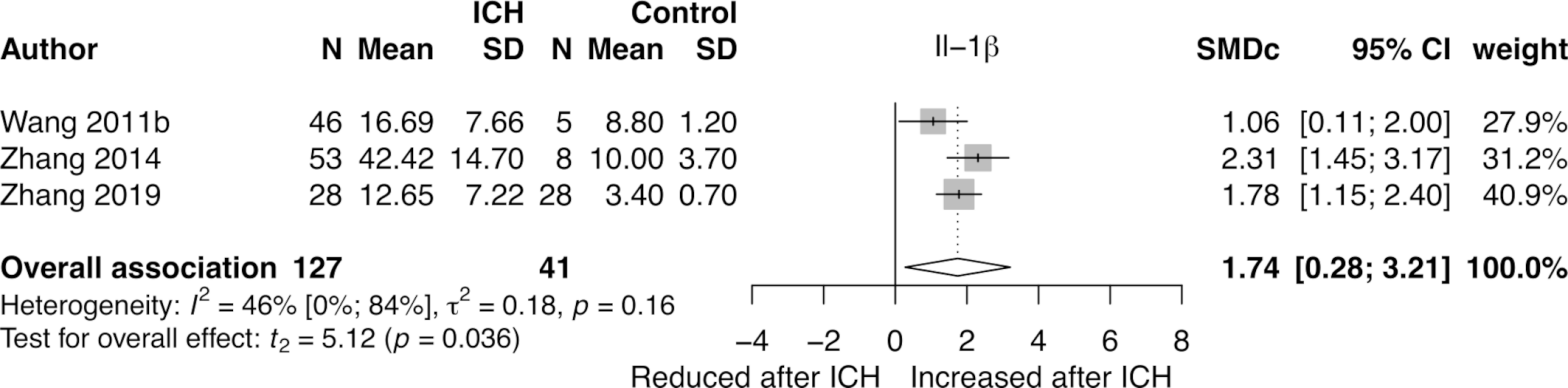
Forest plot of pooled independent associations of IL-1β protein with ICH. Tissue analysed by immunohistochemistry. Studies of surgically resected perihaematomal tissue compared with healthy-appearing tissue that was resected on approach to the haematoma^23^ or from controls undergoing surgery for non-haemorrhagic disease.^22^ ^24^ ICH, intracerebral haemorrhage; IL-1β, interleukin-1β; SMDc, corrected standardised mean difference.

We pooled associations described by these studies of 127 patients and 41 controls using random effects meta-analysis, which showed a statistically significant association of increased IL-1β expression after ICH (SMDc 1.74, 95% CI 0.28 to 3.21; p=0.036; I^2^=46%; figure 3). All studies reported subgroups of patients who underwent surgery at <6 hours and >6 hours after ICH onset. At <6 hours, there was no statistically significant association of IL-1β with ICH (SMDc 2.44, 95% CI −2.76 to 7.64; p=0.18; I^2^=88%; online supplemental figure 1A), whereas at >6 hours after ICH onset, IL-1β was associated with ICH compared with control tissue (SMDc 2.31, 95% CI 0.15 to 4.47; p=0.044; I^2^=70%; online supplemental figure 1B). Funnel plot analysis demonstrated no evidence of publication bias (online supplemental figure 2). Although they did not meet criteria for meta-analysis, four other included studies reported analysis of IL-1β mRNA,^20 21 23 25^ and one used western blotting (WB) to measure IL-1β protein.^23^ All of these analyses found overall IL-1β expression to be increased after ICH compared with control samples. Two studies conducted analysis by time from onset to death. These studies reported that IL-1β mRNA and protein abundance were not increased less than 12 hours after ICH onset but were increased at later time points up to 72 hours.^23 25^

Other molecules were analysed by multiple studies but did not meet criteria for meta-analysis (online supplemental table 6). Subunits of the NF-κB complex were subject to 10 analyses by five studies, which measured mRNA^20 26^ and protein^22 24 26 27^ abundance. Five of six mRNA analyses were by microarray and found no association with NFKB1, NFKB2, REL, RELA or RELB subunits with ICH compared with control tissue.^20^ However, hypothesis-driven analysis of NFKB1 mRNA, by semiquantitative reverse transcription PCR (RT-PCR),^26^ or RELA^24 27^ and multimeric NF-κB complex^22 26^ protein by IHC and WB found positive associations with ICH compared with control tissue. Tumour necrosis factor (TNF)-α was subject to eight analyses by six studies. One microarray analysis found no association of TNF with ICH, whereas other analyses using RT-PCR,^25 26^ quantitative RT-PCR,^23^ IHC^24 25 28^ and WB^23^ found positive associations of TNF with ICH tissue compared with control tissue. Eleven studies used terminal deoxynucleotidyl transferase dUTP nick end labelling (TUNEL) to identify double-stranded DNA breaks, which are associated with apoptosis, and all of these determined that it was increased after ICH (online supplemental table 6).

### GO enrichment analysis

**Figure 4:**
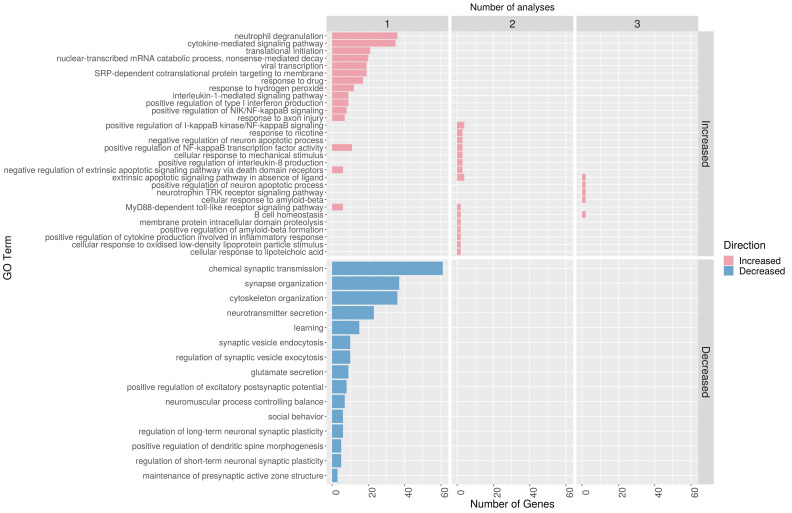
Significantly enriched GO biological process terms according to direction of association with ICH. Top 15 most statistically significantly enriched terms in sets of gene symbols that were subject to at least one, two or three analyses by controlled studies included in our review and found to be increased or decreased after ICH. Ranked by number of genes annotated for each go term. GO, Gene Ontology.

We next sought to identify GO biological processes that were enriched in sets of gene symbols reported by all controlled studies (figure 1 and table 1). GO terms associated with inflammation and suppression of apoptosis were enriched in pooled sets of gene symbols that were found to be increased in one or more studies (figure 4). The most highly enriched were GO:0043066 negative regulation of apoptotic process (enrichment 36/326, p=7×10^−19^) and GO:0043312 neutrophil degranulation (enrichment 36/575, p=1×10^−5^). Conversely, terms associated with neuronal activity were enriched in pooled sets of gene symbols that were found to be reduced in single studies. The most highly enriched of these were GO:0007268 chemical synaptic transmission (enrichment 57/467, p=9×10^−5^) and GO:0050808 synapse organisation (enrichment 31/328, p=1×10^−4^). Gene symbols contributing to enrichment of multiple GO terms are described in online supplemental figures 3 and 4. Full enrichment tables are provided in online supplemental table 8.

**Table 1:** Gene symbols analysed after ICH

Analyses (n)	Gene symbols (n)	
Increased >1.5-fold	mNOS 0–3	mNOS 4–9
≥1 analysis	2	324*
≥2 analyses	0	23*
≥3 analyses	0	10*
≥4 analyses	0	6
≥5 analyses	0	2
Reduced >1.5-fold		
≥1 analysis	0	290*
≥2 analyses	0	1
Unchanged		
≥1 analysis	3	20 785*
≥2 analyses	3	22*
≥3 analyses	0	2

Gene symbols analysed in controlled studies of human brain tissue after ICH according to the number of analyses of each gene symbol and risk of bias.

*Gene sets of >10 genes per set used for Gene Ontology enrichment analysis.

ICH, intracerebral haemorrhage; mNOS, modified Newcastle-Ottawa Scale.

## Discussion

In this systematic review of 38 studies of inflammation in human brain tissue after ICH, we identified analyses of 20 951 molecules of which only one—IL-1β protein—has been analysed more than two times and reported by studies that quantified its association with ICH. IL-1β protein is significantly increased from 6 hours after ICH onset.^22–24^ In addition, we found enrichment of GO terms associated with inflammatory processes, such as negative regulation of apoptotic process and neutrophil degranulation in sets of genes that were increased after ICH.

Our primary finding of increased IL-1β protein >6 hours after ICH is supported by experimental studies which demonstrated that haemorrhage-induced IL-1β expression in the rodent brain was associated worse functional outcome.^29 30^ Because there are substantial differences between human ICH and experimental ICH models, it is critical that inflammatory responses to ICH identified by preclinical studies are shown to be conserved in humans prior to the commencement of clinical trials of immune modulators.^4 5^ An interleukin-1 receptor antagonist is the subject of one ongoing phase II trial for the treatment of ICH,^31^ another which is being prepared (principal investigator: FHBMS), a phase III trial for subarachnoid haemorrhage^32^ and a phase II trial in ischaemic stroke.^33^ The consistent association between ICH and IL-1β expression supports the prioritisation of IL-1β pathway modulators for therapeutic development in ICH.

Our secondary analysis of GO enrichment implicates biological processes that may be activated or suppressed after ICH in humans. In large sets of genes that were increased after ICH, there was enrichment of GO terms associated with neutrophil degranulation and negative regulation of apoptosis. This indicates that neutrophils accumulate in the human brain after ICH and are supported by studies of rodent models of ICH, where rapid neutrophil accumulation contributes to blood–brain barrier dysfunction and neuronal injury.^34 35^ Neutrophils obtained from haematomas drained from patients with ICH exhibit induction of proinflammatory genes, including IL-1β.^36^ Increased peripheral blood neutrophil counts are associated with worse functional outcome at 3 months after ICH.^37^ Eleven studies found increased TUNEL signal after ICH compared with control tissue, indicating increased apoptosis after ICH. We found enrichment of the term negative regulation of apoptosis in our study. Molecules associated with enrichment of this term in our study have previously been associated with processes that control or mitigate ICH-induced apoptosis in rodent models of ICH, including immune cell migration, proliferation and phagocytosis of apoptotic cells.^38^

Our enrichment analyses include sets of genes derived from the findings of many studies, although individual genes may have been subject to few analyses. These analyses generate hypotheses concerning processes associated with ICH, which require testing and validation in human brain tissue. The expression of multiple elements of biological processes may be simultaneously analysed using high-throughput analytical approaches, the availability of which has increased substantially over the past decade.^39^ As such, transcriptomic and proteomic analysis of the human brain after ICH is a priority to enable the rational targeting of specific cells or molecules for therapeutic intervention.

Strengths of our study include the use of a comprehensive search strategy, not restricted by language or date of publication, thorough critical appraisal to determine inclusion and independent data extraction by two reviewers. We identified many analyses of human brain tissue, some of which were not considered by previous narrative reviews of responses to ICH in humans.^3^ We used a stepwise selection process to determine that just three analyses of one marker were reported with sufficient data to permit meta-analysis. These analyses were conducted by three research groups from different institutions.

Our study also has some limitations. Of the 44 included studies, 42 aimed to address a specific hypothesis and selected targets for analysis on that basis. Using prior knowledge to inform selection of target molecules may have driven selective replication and publication of findings that were predicted to show an association with ICH. Moreover, attempts to confirm pre-existing hypotheses may have driven confirmation bias. These may account for the paucity of replicated analyses of gene symbols which were reduced or unchanged after ICH. Just two studies used a hypothesis-free approach, which was RNA microarray.^20 21^ These two studies contributed many gene symbols to our enrichment analyses, which risk introducing biases and imprecision associated with older RNA analysis technologies that are susceptible to type II error to our findings.^40^

## Conclusion

In conclusion, we have determined that IL-1β is consistently increased beyond 6 hours after ICH. Since this is the only marker of brain inflammation which has been reported by more than two studies of human brain tissue after ICH, it should be prioritised as a potential therapeutic target after ICH. We have identified biological processes, including negative regulation of apoptosis and neutrophil degranulation, that are potentially associated with ICH. These require further study to confirm their roles in the neuroinflammatory response after ICH.

## Data Availability

Analysis scripts will be shared on contact with the corresponding author after publication. Gene sets derived from human RNAseq analyses may be available to reasonable requests with appropriate ethical approval. Data for the meta-analysis dataset were extracted from published reports or provided by their corresponding authors.
