# Supplementary material for: Secondary injury and inflammation after intracerebral haemorrhage: a systematic review and meta-analysis of molecular markers in patient brain tissue
Source: J Neurol Neurosurg Psychiatry. 2021 Aug 6;93(2):126–32. doi: 10.1136/jnnp-2021-327098 (PMC8785052; doi:10.1136/jnnp-2021-327098)
Supplement: Supplementary data [file jnnp-2021-327098supp001.pdf]

Secondary injury and inflammation after intracerebral haemorrhage: a systematic review and meta-analysis of molecular markers in patient brain tissue

Authors

James J.M. Loan MSc,[1,2,3] Caoimhe Kirby MSc,[1,2,3] Katherine Emelianova PhD,[2,3] Owen R. Dando PhD,[2,3] Michael T.C. Poon MSc,[1,4] Leisan Pimenova MD,[5] Giles E. Hardingham PhD,[2,3] Barry W. McColl PhD,[2,3] Catharina J.M. Klijn PhD,[6] Rustam Al-Shahi Salman FRCP Edin,[1] Floris H.B.M Schreuder PhD,[6]* Neshika Samarasekera PhD[1]*

Affiliations

1. Centre for Clinical Brain Sciences, University of Edinburgh, Edinburgh, UK
2. Centre for Discovery Brain Sciences, University of Edinburgh, Edinburgh, UK
3. UK Dementia Research Institute at Edinburgh, University of Edinburgh, Edinburgh, UK
4. Usher Institute, University of Edinburgh, Edinburgh, UK
5. München Klinik Bogenhausen, Munich, Germany
6. Department of Neurology, Donders Institute for Brain, Cognition and Behaviour, Radboud University Medical Centre, Nijmegen, Netherlands

*These authors contributed equally

Index of appendices

Supplementary table 1: Search strategies 2
Supplementary table 2: Data extraction items 7
Supplementary table 3: Risk of bias assessment 9
Supplementary table 4: Characteristics of patients used to derive reference genome 10
Supplementary table 5: Characteristics of included studies. 11
Supplementary table 6: Summary of findings. 15
Supplementary table 7: Risk of bias. 19
Supplementary figure 1: Forest plot of pooled independent associations of interleukin-1β protein with ICH stratified by time from ICH onset to tissue retrieval. 24
Supplementary figure 2: Funnel plot of studies included in meta-analysis of associations of interleukin-1β with ICH. 25
Supplementary figure 3: Gene symbols contributing to enrichment of significantly enriched GO biological process terms. 26
Supplementary figure 4: Chord diagrams of significantly enriched GO terms. 27
Supplementary table 8: Enrichment table 29
References for supplementary materials 32

Loan JJM, *et al. J Neurol Neurosurg Psychiatry* 2022; 93:126–132. doi: 10.1136/jnnp-2021-327098

Supplementary table 1: Search strategies

Ovid MEDLINE: 1946-18 June 2020

··	Search terms
1	inflammation/ or inflammat*.mp.
2	immune system.mp. or immune system/
3	cellular immunity/ or innate immunity/ or adaptive immunity/ or immunity/ or humoral immunity/
4	glia cell/ or astrocyte/ or astrocyt*.mp.
5	immunohistochemistry/ or microglia/ or nervous system inflammation/ or microgli*.mp.
6	leukocyte/ or lymphocyte/ or Leukocyt*.mp. or monocyte/
7	glia/ or glia*.mp.
8	neuroglia*.mp.
9	macrophage/ or phagocytosis/ or phagocyte/ or phagocyt*.mp. or neutrophil/
10	(macrophage or neutrophil*).mp.
11	monocyt*.mp. or autoinflammatory disease/
12	granulocyte/ or granulocy*.mp.
13	tanycyte/ or Tanycyte*.mp.
14	necro*.mp.
15	necrosis/ or brain necrosis/
16	hyperemia/ or hyper$emi*.mp.
17	apoptosis/ or apopto*.mp.
18	gliosis/
19	glio*.mp.
20	blood clot lysis/ or clot*.mp. or blood clot/ or blood clot retraction/
21	thrombin/ or thrombosis/
22	tumor necrosis factor alpha/ or interleukin 6/ or cytokine/ or endogenous compound/ or cytokin*.mp. or alpha interferon/ or cytokinesis/
23	chemotaxis/ or chemokinesis/ or chemokine/ or chemokin*.mp.
24	interleukin.mp. or cytokine/
25	(Interleukin?1* or il?1* or Il1* or Interleukin?2* or il?2* or Il2* or Interleukin?3* or il?3* or Il3* or Interleukin?4* or il?4* or Il4* or Interleukin?5* or il?5* or Il5* or Interleukin?6* or il?6* or Il6* or Interleukin?7* or il?7* or Il7* or Interleukin?8* or il?8* or Il8* or CXCL8 or Interleukin?9* or il?9* or Il9* or Interleukin?9*).mp. [mp=title, abstract, original title, name of substance word, subject heading word, floating sub-heading word, keyword heading word, protocol supplementary concept word, rare disease supplementary concept word, unique identifier, synonyms]
26	(Msr1 or Cybb or Lgals3bp or Cst7 or Tlr2 or Nme1 or Rps5 or Ssr4 or P2ry13 or Col27a1 or Elmo1 or Numb or Slc2a5 or Cd244 or Spp1 or Lilrb4 or Hcar2 or Fxyd5 or Il1b or MS4A6A or Gpr65 or Tnf or Srgn or Ifi30 or Cxcl10 or Cd72 or Tspo or Il2rg or Gpr84 or Emp3 or Plaur or Tagln2 or Id2 or Sdc3 or Dab2 or Man2a1 or Psat1 or Map3k8 or Ctsl or Mthfd2 or Cxcl16 or Cd74 or Ctsc or Rnf149 or Smim3 or Cpd or HLA-E or Glrx or Adam9 or Galns or C3ar1 or Atp1a1 or Slamf9 or LILRB4 or Sall1 or Rab31 or Clasp2 or Arap3 or Tln2 or Gpr56 or Il7r or P2ry12 or Slc39a14 or Csmd3 or FCGR3A or Ifit1 or Rpl32 or Rps26 or Iqgap1 or Socs3 or Gbp2 or HLA-DOB or Sult1a1 or Tgfbr1 or Naaa or Plxdc2 or Nuak2 or B4galnt1 or Hmox1 or Ier3 or Ccl2 or CCL13 or St6gal1 or Mgll or Rtp4 or Manf or Klhdc8b or Sbno2 or Cd300lf or Capg or EIF1AX or Srgap2 or Gcnt2 or Ifngr1 or Il10ra or Kif21b or Upk1b or Atp8a2 or Nucb2 or Slc46a3 or Tppp or Klhl24 or Garnl3 or Sft2d2 or Flna or Rasgrp3 or Bank1 or Pcmtd2 or Pcmtd1 or Sbf2 or Cass4 or Slc9a9 or Milr1 or C19orf38 or Tlr12P or Aprt or C5ar1 or Cbfa2t3 or Ccr1 or Ctsh or Cx3cr1v Fkbp5 or Lpin1 or Hpgd or Il15rav IL4R or Lgals9 or Tmed1 or P2rx7 or Ptafr or Ccl7 or Sepp1 or Spint1 or Tnfrsf17 or Top3a or Gpr34 or Ip6k1 or Slc40a1 or Gpr35 or Zdhhc12 or Abhd11 or MS4A6A or Smap2 or Rin2 or Snx29 or Atp13a2 or Ifitm2 or Maged2 or Trem2 or Ncln or Rcbtb2 or Ccdc86 or Rnf169 or Lpp or Dennd6a or A630033H20Rik or Arid5a or Ncaph or Marf1 or Slc2a6 or Fcrl1 or Pilra or Gimap6 or ZNF705A or Rab6b or Eif5a or Tacc1 or Bend6 or Gmppb or Pilrb2).mp. [mp=title, abstract, original title, name of substance word, subject heading word, floating sub-heading word, keyword heading word, protocol supplementary concept word, rare disease supplementary concept word, unique identifier, synonyms]
27	(HMOX1 or NQO1 or SLC7A11 or SRXN1 or GCLC or CAT or NFE2L2).mp. [mp=title, abstract, original title, name of substance word, subject heading word, floating sub-heading word, keyword heading word, protocol supplementary concept word, rare disease supplementary concept word, unique identifier, synonyms]
28	(LCN2 or STEAP4 or S1PR3 or TIMP1 or HSPB1 or CXCL10 or CD44 or OSMR or CP or SERPINA3 or ASPG or VIM or GFAP or C3 or HLA-E or SERPING1 or HLA-A or GBP2 or FBLN5 or FKBP5 or PSMB8 or SRGN or AMIGO2 or CLCF1 or TGM1 or PTX3 or S100A10 or SPHK1 or CD109 or PTGS2 or EMP1 or SLC10A6 or TM4SF1 or B3GNT5 or CD14).mp. [mp=title, abstract, original title, name of substance word, subject heading word, floating sub-heading word, keyword heading word, protocol supplementary concept word, rare disease supplementary concept word, unique identifier, synonyms]
29	NF-kappa B/ or (NF-E2-Related Factor 2 or Nuclear factor erythroid 2-related factor 2 or NRF2).mp. or Heme oxygenase-1/ or HMGB1 protein/ or hirudins/ or (Argatroban or Nafamostat mesilate or Oxymatrine or TAK-242).mp.
30	heme*.mp. or heme oxygenase/ or heme/ or heme oxygenase 1/
31	hemin.mp. or hemin/
32	fibrin*.mp. or fibrin degradation product/ or fibrin/
33	superoxide.mp. or superoxide/

Loan JJM, *et al. J Neurol Neurosurg Psychiatry* 2022; 93:126–132. doi: 10.1136/jnnp-2021-327098

34	hydrogen peroxide.mp. or hydrogen peroxide/
35	H2O2.mp.
36	complement component C2a/ or complement component C2b/ or complement factor D/ or complement receptor antagonist/ or complement component C1r/ or complement membrane attack complex/ or complement activation/ or complement component C5a/ or complement/ or complement component C5a receptor/ or complement component C1/ or complement component C4b binding protein/ or complement component C6/ or complement system/ or complement inhibitor/ or classical complement pathway C3 C5 convertase/ or complement component C4d/ or complement component C8b/ or complement component C3d receptor/ or complement dependent cytotoxicity/ or complement component C4 binding protein/ or complement inhibition/ or complement component C3/ or complement component C2/ or complement component C3 receptor/ or complement component C3a/ or complement factor I/ or complement component C9/ or complement component C5 inhibitor/ or complement factor H/ or complement component C8/ or alternative complement pathway C3 C5 convertase/ or complement fixation/ or complement component C3c/ or complement.mp. or complement blood level/ or complement component C1q antibody/ or complement deposition/ or complement component C4b/ or complement alternative pathway/ or complement component C3b receptor/ or vaccinia virus complement control protein/ or complement component C1s inhibitor/ or complement component C3b/ or complement component C5/ or complement receptor/ or complement component C4/ or complement component C8a/ or complement component C1s/ or complement component C4a/ or complement factor/ or complement classical pathway/ or "complement component C5a [dearginine]"/ or complement component C3 inhibitor/ or complement component C3d/ or complement component C7/ or complement component C5b/ or complement component C5a receptor antagonist/ or complement receptor affecting agent/ or complement component C1q/ or complement fixation test/
37	interferon.mp. or interferon/
38	NF$KB.mp.
39	tumor necrosis factor.mp.
40	matrix metalloproteinase/ or matrix metalloproteinase inhibitor/ or metalloproteinase/ or matrix metalloprotease.mp. or collagenase/
41	MMP*.mp.
42	AQ$4.mp. or aquaporin 4/
43	aquaporin?4.mp.
44	NOS.mp.
45	nitric oxide synthase/
46	caspase inhibitor/ or caspase 5/ or caspase 8 inhibitor/ or initiator caspase/ or caspase 12/ or caspase 11/ or caspase 6/ or caspase 10/ or caspase 14/ or caspase/ or caspase recruitment domain protein 4/ or apoptosis repressor with caspase recruitment domain/ or caspase 9 inhibitor/ or caspase.mp. or caspase 13/ or caspase recruitment domain signaling protein/ or caspase 8/ or caspase 2 inhibitor/ or caspase 3/ or caspase activated deoxyribonuclease/ or caspase recruitment domain protein 15/ or "second mitochondrial activator of caspase"/ or effector caspase/ or caspase 3 inhibitor/ or caspase 9/ or caspase 4/ or "caspase activation and recruitment domain"/ or caspase assay/ or caspase 2/ or caspase 7/
47	Damage associated molecular pattern.mp.
48	DAMP.mp.
49	pathogen associated molecular pattern/ or PAMP.mp.
50	autophagy.mp. or autophagy/
51	toll like receptor 4/ or toll$like receptor.mp. or toll like receptor/
52	toll like receptor 2/
53	inflammasome/ or inflammasone.mp.
54	eicosanoid.mp. or icosanoid/
55	leukotriene D4/ or leukotriene D4 derivative/ or leukotriene A4 hydrolase inhibitor/ or leukotriene A4 methyl ester/ or leukotriene B4/ or leukotriene A4 derivative/ or leukotriene receptor affecting agent/ or leukotriene A4 hydrolase/ or leukotriene B3/ or leukotriene/ or leukotriene E4/ or leukotriene receptor blocking agent/ or leukotriene receptor stimulating agent/ or leukotriene.mp. or "prostaglandin,thromboxane or leukotriene receptor affecting agents"/ or leukotriene A4/ or leukotriene B4 receptor/ or leukotriene B5/ or leukotriene derivative/ or leukotriene receptor/ or leukotriene E4 derivative/ or leukotriene C4 derivative/ or leukotriene B4 receptor antagonist/ or leukotriene C4/ or leukotriene F4/ or leukotriene C4 synthase/ or leukotriene B4 derivative/ or leukotriene D4 receptor/
56	prostaglandin F1 alpha/ or prostaglandin F2/ or prostaglandin blood level/ or prostaglandin E synthase 1/ or prostaglandin E receptor 2/ or prostaglandin D synthase/ or delta12 prostaglandin J2/ or prostaglandin/ or prostaglandin A/ or prostaglandin E3/ or prostaglandin synthesis/ or prostaglandin synthesis inhibition/ or prostaglandin E2 trometamol/ or prostaglandin E synthase/ or prostaglandin E receptor 1/ or prostaglandin E1 derivative/ or prostaglandin receptor/ or "prostaglandin,thromboxane or leukotriene receptor affecting agents"/ or prostaglandin B1 polymer/ or prostaglandin synthase/ or prostaglandin B/ or prostaglandin A2 isopropyl ester/ or prostaglandin B2/ or prostaglandin E receptor/ or prostaglandin F/ or prostaglandin endoperoxide/ or prostaglandin G/ or prostaglandin F2 alpha/ or prostaglandin G2/ or prostaglandin B1/ or prostaglandin receptor stimulating agent/ or prostaglandin D/ or 15 deoxy delta12,14 prostaglandin J2/ or prostaglandin urine level/ or prostaglandin derivative/ or prostaglandin J2/ or prostaglandin E2 derivative/ or prostaglandin E receptor 4/ or prostaglandin D2 derivative/ or prostaglandin metabolism/ or prostaglandin D2/ or prostaglandin H/ or prostaglandin E2/ or prostaglandin F2 alpha trometamol/ or prostaglandin H2/ or prostaglandin receptor blocking agent/ or prostaglandin F2 alpha isopropyl ester/ or prostaglandin synthase inhibitor/ or prostaglandin A1/ or prostaglandin E1/ or prostaglandin inhibitor/ or prostaglandin F3 alpha/ or prostaglandin E/ or prostaglandin B1 derivative/ or prostaglandin F2 alpha derivative/ or prostaglandin E2 methyl ester/ or prostaglandin I3/ or prostaglandin transporter/ or prostaglandin A2/ or prostaglandin release/ or prostaglandin.mp. or prostaglandin E receptor 3/ or prostaglandin receptor affecting agent/
57	prostacyclin/

58	((brain$ or cerebr$ or cerebell$ or intracerebr$ or intracran$ or parenchyma$ or intraventricular or infratentorial or supratentorial or basal gang$ or ganglion$ or putaminal or putamen or posterior fossa or brain?stem or intra?axial or lobar or deep or thalam$ or cortical or superficial or vertebrobasil$ or front$ or tempor$ or pariet$ or occipit$) adj (haemorrhage$ or hemorrhage$ or haematoma$ or hematoma$ or bleed$)).tw.
59	(h?emorrhag$ adj (stroke$ or cerebrovasc$ or cerebr?vasc$ or cerebral vasc$ or brain vasc$ or cva$ or apoplex$ or attack$ or event$ or insult$)).tw.
60	basal ganglion hemorrhage/ or exp brain hematoma/ or exp brain hemorrhage/ or cerebral hemorrhage/
61	58 or 59 or 60
62	Receptors, Cell Surface/
63	(post$mortem or biops* or autopsy or intra$operative or patholog* or histopatholog*).mp. or immunohistochemistry/
64	BRAIN/en [Enzymology]
65	62 or 63 or 64
66	1 or 2 or 3 or 4 or 5 or 6 or 7 or 8 or 9 or 10 or 11 or 12 or 13 or 14 or 15 or 16 or 17 or 18 or 19 or 20 or 21 or 22 or 23 or 24 or 25 or 26 or 27 or 28 or 29 or 30 or 31 or 32 or 33 or 34 or 35 or 36 or 37 or 38 or 39 or 40 or 41 or 42 or 43 or 44 or 45 or 46 or 47 or 48 or 49 or 50 or 51 or 52 or 53 or 54 or 55 or 56 or 57
67	(t?cell or t?lymphocyt* or t-cell).mp. or T lymphocyte/
68	66 or 67
69	61 and 65 and 68
70	exp animals/ not humans/
71	69 not 70

Ovid EMBASE: 1974 to 18 June 2020

..	**Search terms**
1	inflammation/ or inflammat*.mp.
2	immune system.mp. or immune system/
3	cellular immunity/ or innate immunity/ or adaptive immunity/ or immunity/ or humoral immunity/
4	glia cell/ or astrocyte/ or astrocyt*.mp.
5	immunohistochemistry/ or microglia/ or nervous system inflammation/ or microgli*.mp.
6	leukocyte/ or lymphocyte/ or Leukocyt*.mp. or monocyte/
7	glia/ or glia*.mp.
8	neuroglia*.mp.
9	macrophage/ or phagocytosis/ or phagocyte/ or phagocyt*.mp. or neutrophil/
10	(macrophage or neutrophil*).mp.
11	monocyt*.mp. or autoinflammatory disease/
12	granulocyte/ or granulocy*.mp.
13	tanycyte/ or Tanycyte*.mp.
14	necro*.mp.
15	necrosis/ or brain necrosis/
16	hyperemia/ or hyper$emi*.mp.
17	apoptosis/ or apopto*.mp.
18	gliosis/
19	glio*.mp.
20	blood clot lysis/ or clot*.mp. or blood clot/ or blood clot retraction/
21	thrombin/ or thrombosis/
22	tumor necrosis factor alpha/ or interleukin 6/ or cytokine/ or endogenous compound/ or cytokin*.mp. or alpha interferon/ or cytokinesis/
23	chemotaxis/ or chemokinesis/ or chemokine/ or chemokin*.mp.
24	interleukin.mp. or cytokine/
25	(Interleukin?1* or il?1* or Il1* or Interleukin?2* or il?2* or Il2* or Interleukin?3* or il?3* or Il3* or Interleukin?4* or il?4* or Il4* or Interleukin?5* or il?5* or Il5* or Interleukin?6* or il?6* or Il6* or Interleukin?7* or il?7* or Il7* or Interleukin?8* or il?8* or Il8* or CXCL8 or Interleukin?9* or il?9* or Il9* or Interleukin?9*).mp. [mp=title, abstract, heading word, drug trade name, original title, device manufacturer, drug manufacturer, device trade name, keyword, floating subheading word, candidate term word]
26	(Msr1 or Cybb or Lgals3bp or Cst7 or Tlr2 or Nme1 or Rps5 or Ssr4 or P2ry13 or Col27a1 or Elmo1 or Numb or Slc2a5 or Cd244 or Spp1 or Lilrb4 or Hcar2 or Fxyd5 or Il1b or MS4A6A or Gpr65 or Tnf or Srgn or Ifi30 or Cxcl10 or Cd72 or Tspo or Il2rg or Gpr84 or Emp3 or Plaur or Tagln2 or Id2 or Sdc3 or Dab2 or Man2a1 or Psat1 or Map3k8 or Ctsl or Mthfd2 or Cxcl16 or Cd74 or Ctsc or Rnf149 or Smim3 or Cpd or HLA-E or Glrx or Adam9 or Galns or C3ar1 or Atp1a1 or Slamf9 or LILRB4 or Sall1 or Rab31 or Clasp2 or Arap3 or Tln2 or Gpr56 or Il7r or P2ry12 or Slc39a14 or Csmd3 or FCGR3A or Ifit1 or Rpl32 or Rps26 or Iqgap1 or Socs3 or Gbp2 or HLA-DOB or Sult1a1 or Tgfbr1 or Naaa or Plxdc2 or Nuak2 or B4galnt1 or Hmox1 or Ier3 or Ccl2 or CCL13 or St6gal1 or Mgll or Rtp4 or Manf or Klhdc8b or Sbno2 or Cd300lf or Capg or EIF1AX or Srgap2 or Gcnt2 or Ifngr1 or Il10ra or Kif21b or Upk1b or Atp8a2 or Nucb2 or Slc46a3 or Tppp or Klhl24 or Garnl3 or Sft2d2 or Flna or Rasgrp3 or Bank1 or Pcmtd2 or Pcmtd1 or Sbf2 or Cass4 or Slc9a9 or Milr1 or C19orf38 or Tlr12P or Aprt or C5ar1 or Cbfa2t3 or Ccr1 or Ctsh or Cx3cr1v Fkbp5 or Lpin1 or Hpgd or Il15rav IL4R or Lgals9 or Tmed1 or P2rx7 or Ptafr or Ccl7 or Sepp1 or Spint1 or Tnfrsf17 or Top3a or Gpr34 or Ip6k1 or Slc40a1 or Gpr35 or Zdhhc12 or Abhd11 or

	MS4A6A or Smap2 or Rin2 or Snx29 or Atp13a2 or Ifitm2 or Maged2 or Trem2 or Ncln or Rcbtb2 or Ccdc86 or Rnf169 or Lpp or Dennd6a or A630033H20Rik or Arid5a or Ncaph or Marf1 or Slc2a6 or Fcrl1 or Pilra or Gimap6 or ZNF705A or Rab6b or Eif5a or Tacc1 or Bend6 or Gmppb or Pilrb2).mp. [mp=title, abstract, heading word, drug trade name, original title, device manufacturer, drug manufacturer, device trade name, keyword, floating subheading word, candidate term word]
27	(HMOX1 or NQO1 or SLC7A11 or SRXN1 or GCLC or CAT or NFE2L2).mp. [mp=title, abstract, heading word, drug trade name, original title, device manufacturer, drug manufacturer, device trade name, keyword, floating subheading word, candidate term word]
28	(LCN2 or STEAP4 or S1PR3 or TIMP1 or HSPB1 or CXCL10 or CD44 or OSMR or CP or SERPINA3 or ASPG or VIM or GFAP or C3 or HLA-E or SERPING1 or HLA-A or GBP2 or FBLN5 or FKBP5 or PSMB8 or SRGN or AMIGO2 or CLCF1 or TGM1 or PTX3 or S100A10 or SPHK1 or CD109 or PTGS2 or EMP1 or SLC10A6 or TM4SF1 or B3GNT5 or CD14).mp. [mp=title, abstract, heading word, drug trade name, original title, device manufacturer, drug manufacturer, device trade name, keyword, floating subheading word, candidate term word]
29	NF-kappa B/ or (NF-E2-Related Factor 2 or Nuclear factor erythroid 2-related factor 2 or NRF2).mp. or Heme oxygenase-1/ or HMGB1 protein/ or hirudins/ or (Argatroban or Nafamostat mesilate or Oxymatrine or TAK-242).mp.
30	heme*.mp. or heme oxygenase/ or heme/ or heme oxygenase 1/
31	hemin.mp. or hemin/
32	fibrin*.mp. or fibrin degradation product/ or fibrin/
33	superoxide.mp. or superoxide/
34	hydrogen peroxide.mp. or hydrogen peroxide/
35	H2O2.mp.
36	complement component C2a/ or complement component C2b/ or complement factor D/ or complement receptor antagonist/ or complement component C1r/ or complement membrane attack complex/ or complement activation/ or complement component C5a/ or complement/ or complement component C5a receptor/ or complement component C1/ or complement component C4b binding protein/ or complement component C6/ or complement system/ or complement inhibitor/ or classical complement pathway C3 C5 convertase/ or complement component C4d/ or complement component C8b/ or complement component C3d receptor/ or complement dependent cytotoxicity/ or complement component C4 binding protein/ or complement inhibition/ or complement component C3/ or complement component C2/ or complement component C3 receptor/ or complement component C3a/ or complement factor I/ or complement component C9/ or complement component C5 inhibitor/ or complement factor H/ or complement component C8/ or alternative complement pathway C3 C5 convertase/ or complement fixation/ or complement component C3c/ or complement.mp. or complement blood level/ or complement component C1q antibody/ or complement deposition/ or complement component C4b/ or complement alternative pathway/ or complement component C3b receptor/ or vaccinia virus complement control protein/ or complement component C1s inhibitor/ or complement component C3b/ or complement component C5/ or complement receptor/ or complement component C4/ or complement component C8a/ or complement component C1s/ or complement component C4a/ or complement factor/ or complement classical pathway/ or "complement component C5a [deարginine]"/ or complement component C3 inhibitor/ or complement component C3d/ or complement component C7/ or complement component C5b/ or complement component C5a receptor antagonist/ or complement receptor affecting agent/ or complement component C1q/ or complement fixation test/
37	interferon.mp. or interferon/
38	NF$KB.mp.
39	tumor necrosis factor.mp.
40	matrix metalloproteinase/ or matrix metalloproteinase inhibitor/ or metalloproteinase/ or matrix metalloprotease.mp. or collagenase/
41	MMP*.mp.
42	AQ$4.mp. or aquaporin 4/
43	aquaporin?4.mp.
44	NOS.mp.
45	nitric oxide synthase/
46	caspase inhibitor/ or caspase 5/ or caspase 8 inhibitor/ or initiator caspase/ or caspase 12/ or caspase 11/ or caspase 6/ or caspase 10/ or caspase 14/ or caspase/ or caspase recruitment domain protein 4/ or apoptosis repressor with caspase recruitment domain/ or caspase 9 inhibitor/ or caspase.mp. or caspase 13/ or caspase recruitment domain signaling protein/ or caspase 8/ or caspase 2 inhibitor/ or caspase 3/ or caspase activated deoxyribonuclease/ or caspase recruitment domain protein 15/ or "second mitochondrial activator of caspase"/ or effector caspase/ or caspase 3 inhibitor/ or caspase 9/ or caspase 4/ or "caspase activation and recruitment domain"/ or caspase assay/ or caspase 2/ or caspase 7/
47	Damage associated molecular pattern.mp.
48	DAMP.mp.
49	pathogen associated molecular pattern/ or PAMP.mp.
50	autophagy.mp. or autophagy/
51	toll like receptor 4/ or toll$like receptor.mp. or toll like receptor/
52	toll like receptor 2/
53	inflammasome/ or inflammasone.mp.
54	eicosanoid.mp. or icosanoid/
55	leukotriene D4/ or leukotriene D4 derivative/ or leukotriene A4 hydrolase inhibitor/ or leukotriene A4 methyl ester/ or leukotriene B4/ or leukotriene A4 derivative/ or leukotriene receptor affecting agent/ or leukotriene A4 hydrolase/ or leukotriene B3/ or leukotriene/ or leukotriene E4/ or leukotriene receptor blocking agent/ or leukotriene receptor stimulating agent/ or leukotriene.mp. or "prostaglandin,thromboxane or leukotriene receptor affecting agents"/ or

	leukotriene A4/ or leukotriene B4 receptor/ or leukotriene B5/ or leukotriene derivative/ or leukotriene receptor/ or leukotriene E4 derivative/ or leukotriene C4 derivative/ or leukotriene B4 receptor antagonist/ or leukotriene C4/ or leukotriene F4/ or leukotriene C4 synthase/ or leukotriene B4 derivative/ or leukotriene D4 receptor/
56	prostaglandin F1 alpha/ or prostaglandin F2/ or prostaglandin blood level/ or prostaglandin E synthase 1/ or prostaglandin E receptor 2/ or prostaglandin D synthase/ or delta12 prostaglandin J2/ or prostaglandin/ or prostaglandin A/ or prostaglandin E3/ or prostaglandin synthesis/ or prostaglandin synthesis inhibition/ or prostaglandin E2 trometamol/ or prostaglandin E synthase/ or prostaglandin E receptor 1/ or prostaglandin E1 derivative/ or prostaglandin receptor/ or "prostaglandin,thromboxane or leukotriene receptor affecting agents"/ or prostaglandin B1 polymer/ or prostaglandin synthase/ or prostaglandin B/ or prostaglandin A2 isopropyl ester/ or prostaglandin B2/ or prostaglandin E receptor/ or prostaglandin F/ or prostaglandin endoperoxide/ or prostaglandin G/ or prostaglandin F2 alpha/ or prostaglandin G2/ or prostaglandin B1/ or prostaglandin receptor stimulating agent/ or prostaglandin D/ or 15 deoxy delta12,14 prostaglandin J2/ or prostaglandin urine level/ or prostaglandin derivative/ or prostaglandin J2/ or prostaglandin E2 derivative/ or prostaglandin E receptor 4/ or prostaglandin D2 derivative/ or prostaglandin metabolism/ or prostaglandin D2/ or prostaglandin H/ or prostaglandin E2/ or prostaglandin F2 alpha trometamol/ or prostaglandin H2/ or prostaglandin receptor blocking agent/ or prostaglandin F2 alpha isopropyl ester/ or prostaglandin synthase inhibitor/ or prostaglandin A1/ or prostaglandin E1/ or prostaglandin inhibitor/ or prostaglandin F3 alpha/ or prostaglandin E/ or prostaglandin B1 derivative/ or prostaglandin F2 alpha derivative/ or prostaglandin E2 methyl ester/ or prostaglandin I3/ or prostaglandin transporter/ or prostaglandin A2/ or prostaglandin release/ or prostaglandin.mp. or prostaglandin E receptor 3/ or prostaglandin receptor affecting agent/
57	prostacyclin/
58	((brain$ or cerebr$ or cerebell$ or intracerebr$ or intracran$ or parenchyma$ or intraventricular or infratentorial or supratentorial or basal gang$ or ganglion$ or putaminal or putamen or posterior fossa or brain?stem or intra?axial or lobar or deep or thalam$ or cortical or superficial or vertebrobasil$ or front$ or tempor$ or pariet$ or occipit$) adj (haemorrhage$ or hemorrhage$ or haematoma$ or hematoma$ or bleed$)).tw.
59	(h?emorrhag$ adj (stroke$ or cerebrovasc$ or cerebr?vasc$ or cerebral vasc$ or brain vasc$ or cva$ or apoplex$ or attack$ or event$ or insult$)).tw.
60	basal ganglion hemorrhage/ or exp brain hematoma/ or exp brain hemorrhage/ or cerebral hemorrhage/
61	58 or 59 or 60
62	Receptors, Cell Surface/
63	(post$mortem or biops* or autopsy or intra$operative or patholog* or histopatholog*).mp. or immunohistochemistry/
64	[BRAIN/en [Enzymology]]
65	62 or 63 or 64
66	1 or 2 or 3 or 4 or 5 or 6 or 7 or 8 or 9 or 10 or 11 or 12 or 13 or 14 or 15 or 16 or 17 or 18 or 19 or 20 or 21 or 22 or 23 or 24 or 25 or 26 or 27 or 28 or 29 or 30 or 31 or 32 or 33 or 34 or 35 or 36 or 37 or 38 or 39 or 40 or 41 or 42 or 43 or 44 or 45 or 46 or 47 or 48 or 49 or 50 or 51 or 52 or 53 or 54 or 55 or 56 or 57
67	(t?cell or t?lymphocyt* or t-cell).mp. or T lymphocyte/
68	66 or 67
69	61 and 65 and 68
70	exp animals/ not humans/
71	69 not 70

Supplementary table 2: Data extraction items

**Identification**
Author's name
Institution
Email
Address
Year of Publication
Title
Sponsorship source
Country
Setting
Comments
**Methods**
Design (case series, case control)
Blinding
Comparator disease condition
Comparator group (internal control, external control, uncontrolled, can't tell)
Method for radiological measurement of ICH volume (manual, semiautomated, fully automated)
Method for radiological measurement of PHO volume (manual, semiautomated, fully automated)
Primary outcome
Other relevant outcomes
Principal tissue analysis methods used (frozen, fixed, cell suspension, IHC, IF, qPCR, etc)
**Population**
inclusion criteria
Exclusion criteria
Group differences
Inclusion period and mid-year of study
**Baseline characteristics**
Characteristic
Number of patients
% male
Age range
Age mean +/- SD
Age median +/- IQR
Time from onset to tissue retrieval/death: range (specify units)
Time from onset to tissue retrieval mean +/- SD (specify units)
Time from onset to tissue retrieval median +/- IQR (specify units)
Time from death to post-mortem: range (hours, if applicable)
Time from death to post-mortem: mean +/- SD (hours, if applicable)
Time from death to post-mortem: Median +/- IQR (hours, if applicable)
Number of technical replicates per sample per analysis
Ethnicity: n White
Ethnicity: n Black
Ethnicity: n East Asian
Ethnicity: n Indian
Ethnicity: n Arab
Ethnicity: n Hispanic
Ethnicity: n mixed
Ethnicity: n other
Number of patients using immunomodulatory drugs
**Interventions**
Characteristic
Diagnostic investigation for ICH

Loan JJM, *et al. J Neurol Neurosurg Psychiatry* 2022; 93:126–132. doi: 10.1136/jnnp-2021-327098

Ancillary investigations to rule out secondary cause
Tissue source (post-mortem/biopsy/other)
PHO volumes range
PHO volume mean +/- SD
PHO volume median +/- IQR
ICH volumes range
ICH volumes mean +/-SD
ICH volumes median +/- IQR
SVD diagnosis numbers per group
SVD severity summary measures per group
Unclear location of ICH
Lobar location of ICH
Deep location of ICH
Cerebellar location of ICH
Mixed infratentorial location of ICH
mixed supratentorial location of ICH
CAA cases per group: number diagnosed
Summary CAA severity measure per group
Intraventricular haemorrhage present on initial CT imaging: specify numbers where "yes"
Hydrocephalus present on initial CT imaging: specify numbers where "yes"
Cardiovascular comorbidity prior to presentation (specify number of patients per condition)
Number of cases with cause of death other than ICH. Specific number per cause of death
Number of cases with intervening brain illness between ICH and tissue sampling. Specify numbers per condition
ICH score: range
ICH score mean +/- SD
ICH score median: +/- IQR
NIHSS score range
NIHSS score mean +/- SD
NIHSS score mean +/- IQR
Brainstem location of ICH
GCS: median +/- IQR
GCS: Mean +/- SD
GCS range
First ever ICH (n)
Recurrent ICH (n)
**Outcome:**
For each group, specify each outcome measure for each tissue type as a new line. Enter the data as a Mean +/- SD, Median +/- IQR or Range per group as well as numbers of cases per group

Loan JJM, *et al. J Neurol Neurosurg Psychiatry* 2022; 93:126–132. doi: 10.1136/jnnp-2021-327098

Supplementary table 3: Risk of bias assessment

Risk of Bias	Point awarded if selected
**Selection**	
**1) Is case definition adequate?**	
a) Yes with independent validation	1
b) Yes, e.g. record linkage or based on self-reports	0
c) no description	0
**2) Representativeness of the cases**	
a) consecutive or obviously representative series of cases *	1
b) potential for selection biases or not stated	0
**3) Selection of controls**	
a) community controls*	1
b) tissue from same person as case but distant to ICH*	1
b) hospital controls	0
c) no description	0
**4) Definition of controls**	
a) no history of disease (endpoint)*	1
b) distant brain tissue	0
c) no description of source/Other	0
**Comparability**	
**1) comparability of cases and controls on the basis of design or analysis**	
a) study controls for .... (Select the most important factor)*	1
b) study controls for any additional factor *	1
**2) Were all tissues processed in the same manner?**	
a) all tissues processed in the same manner with equal numbers of cases and controls per batch*	1
b) tissues processed using the same technique but potential confounders affecting either case or control tissue	0
c) tissue processing of cases and controls was different	0
**Exposure**	
**1) same method of case ascertainment for cases and controls**	
a) yes*	1
b) no	0
c) unclear	0
**2) Were all cases and controls accounted for in all analyses**	
a) data from all cases and controls provided*	1
b) data missing but an explanation for missing data provided	0
c) unclear if all data provided or data missing without explanation	0

Loan JJM, *et al. J Neurol Neurosurg Psychiatry* 2022; 93:126–132. doi: 10.1136/jnnp-2021-327098

Supplementary table 4: Characteristics of patients used to derive reference genome

ID	Age	Sex	Death-autopsy interval (h)	RIN*	Neuropathological findings	Cause of death
1	89	female	24	6.1	No abnormality detected	Cardiac failure
2	76	female	129.5	7.3	No abnormality detected	Ischaemic heart disease
3	71	female	96	6.6	No abnormality detected	Ischaemic heart disease
4	57	male	64	7.3	Mild non-amyloid SVD	Ischaemic heart disease
5	71	female	95	5.8	Mild non-amyloid SVD	Suffocation
6	72	male	60	6.3	Mild non-amyloid SVD	Ischaemic heart disease
7	73	male	66	5.9	Moderate non-amyloid SVD, mild arteriolar Aß-CAA, Braak tangle stage I	Ischaemic heart disease

*RIN: RNA integrity number.

Loan JJM, et al. J Neurol Neurosurg Psychiatry 2022; 93:126–132. doi: 10.1136/jnnp-2021-327098

Supplementary table 5: Characteristics of included studies.

Control brain tissue from the same case as the ICH was termed *internal controls* and control tissue derived from unaffected individuals was termed *external controls*. † included in final meta-analysis dataset; PMI – death to post-mortem interval; IC – internal control; EC – external control; NS – not stated; NA – not applicable; *mean±standard deviation; **mean; *** range; external control – control tissue from a different patient to case; internal control – control tissue from same patient as case; h – hours; d – days

Study	Country / Ethnicity	Tissue source	Control type (IC/EC)	ICH location	Time from ICH onset to biopsy / death	Cases				Controls			
						n	Age in years	% Male	PMI	n	Age in years	% Male	PMI
Bao 2011a[1]†	China / NS	Biopsy	Distant tissue from unclear hemisphere (IC)	NS	NS	14	NS	NS	NA	14	NS	NS	NA
Bao 2011b[2]†	China / NS	Biopsy	Tissue from middle cerebral gyrus of unclear hemisphere (IC)	NS	NS	31	NS	NS	NA	31	NS	NS	NA
Camacho 2019[3]	Spain / NS	Post-mortem	Patients with cerebral amyloid angiopathy but no ICH (EC)	NS	NS	7	83±6*	43	2-20h	7	73±11*	43	2-20h
Carmichael 2008[4]	USA / ICH: 3 Caucasian, 1 Arab and 1 Hispanic ICH. Control: 5 Caucasian	Biopsy	Non-anatomically matched post-mortem “healthy aged“ brain tissue (EC)	Lobar 4, deep 2	18±7·5h*	6	69±12*	NS	NA	5	79±3*	NS	3±1h
Chen 2008[5]†	China / NS	Biopsy	Distant tissue from unclear hemisphere (IC)	Lobar 3, deep 25, infratentorial 2	<6h to >72h	30	55**	70	NA	7	56**	71	NA
Dahnovici 2011[6]	Romania / NS	Post-mortem	Contralateral tissue (IC)	NS	NS	24	68-83	NS	NS	24	68-83	NS	NS
Delgado 2008[7]	Spain / NS	Post-mortem	Contralateral tissue and tissue from patients with no ICH, inflammatory, or neurological disease (IC & EC)	NS	23±15h*	6	79±6*	83	<6h	IC 6, EC 2	IC 79±6, EC 73±9*	IC 83, EC 50	<6h
Di Napoli 2012[8]	Romania / NS	Post-mortem	Contralateral brain tissue and tissue from patients with no brain pathology (IC & EC)	NS	4-12h	5	74±3*	0	4-8h	IC 5, EC 2	IC 74±3, EC 81±2*	IC 0, EC 50	4-8h
Duan 2007[9]	China / NS	Biopsy	Contralateral brain tissue (IC)	Lobar 21, deep 18	112h**	39	63**	44	NA	39	63**	44	NA
Gang 2018[10]	China / NS	Biopsy	Brain tissue >1cm distant from ICH (IC)	Supratentorial	6 patients <6h, 6 patients 6-24h, 6 patients 24-27h, 6 patients >72h	24	60±16*	67	NA	24	60±16*	67	NA
Guo 2008[11]†	China / NS	Biopsy	Normal appearing tissue taken on surgical approach to haematoma (IC)	Lobar 3, Deep 25, Cerebellar 2	NS	30	55**	70	NA	7	NS	NS	NA

Study	Country / Ethnicity	Tissue source	Control type (IC/EC)	ICH location	Time from ICH onset to biopsy / death	Cases				Controls			
						n	Age in years	% Male	PMI	n	Age in years	% Male	PMI
Hernandez-Guillamon 2012[12]	Spain / NS	Post-mortem	Contralateral brain tissue and tissue from patients who died of cardiorenal failure, gastrointestinal haemorrhage or legionellosis (EC & IC)	Lobar 4, deep 5,	<4->96h	9	83±8*	44	<6h	IC 9, EC 3	IC 83±8, EC 72±10*	IC 44, EC 33	<6h
Holfelder 2011[13]	Germany / NS	Post-mortem	Distant tissue from unclear location and tissue from "corresponding locations" of "unaffected brains" (EC & IC)	NS	225±285h*	12	60±14*	58	NS	IC 12, EC 6	IC 60±14, EC 50±21*	IC 58, EC 50	NS
Itoh 1997[14]	Japan / NS	Post-mortem	Uncontrolled	NS	NS	15	NS	NS	NS	0	NA	NA	NA
Jin, 2011[15]	USA / NS	Cases – biopsy; controls – NS	Unclear origin of control tissue (EC)	Deep	2±1d*	5	58±10*	40	NA	4	NS	NS	NS
Ke 2007[16]	China / NS	Biopsy	Uncontrolled	Lobar 9, Deep 33	NS	42	60±1*	64	NA	0	NA	NA	NA
Li, 2010[17]	China / NS	Post-mortem	Contralateral and distant ipsilateral brain tissue (IC)	Lobar 10, deep 22, infratentorial 12	2h-16d	44	60** Range 37-84	59	<12h	44	60** Range 37-84	59	<12h
Liu 2015[18]	China / NS	Biopsy	Distant tissue >1cm from haematoma (IC)	Lobar 8, Deep 19	11 patients <6h, 8 patients 6-24h, 4 patients 24-72h, 4 patients >72h	27	58±13*	67	NA	NS	NS	NS	NA
Liu 2006[19]	China / NS	Biopsy	Uncontrolled	Deep	28±15*	32	56±10*	66	NA	0	NA	NA	NA
Mantle 2001[20]†	UK / NS	Biopsy	Patients surgically treated for brain aneurysm or tumour (EC)	NS	NS	10	NS	NS	NA	6	NS	NS	NA
McCarron 1997[21]	UK / NS	Post-mortem	Tissue from patients with no neuropathologic diagnosis (EC)	NS	NS	37	71**	24	NS	12	76**	45	NS
Rosell 2011[22]†	Spain / NS	Post-mortem	Contralateral brain tissue (IC)	NS	31±30h*	8	80±11.0*	75	6±5h*	8	80±11.0*	75	6±5h*
Rosell 2006[23]	Spain / NS	Post-mortem	Contralateral brain tissue and tissue from patients who died of non-inflammatory disease (EC & IC)	NS	14±8h*	8	79±8*	88	5±1h*	IC 8, EC2	IC 79±8, EC 73±9*	IC 88, EC 50	5±1h*
Shen 2008[24]	China / NS	Biopsy	Uncontrolled	Deep	2±1d*	5	58±10*	40	NA	0	NA	NA	NA
Shtaya 2019[25]	UK / NS	Post-mortem	Brain tissue distant to the ICH and "healthy control" brain tissue (EC & IC)	Lobar 15, deep 12	Not stated for 8/27 cases. 0-12 days	27	63±20*	48	NS	IC 27, EC unclear 16-18	IC 63±20*, EC 26-60***	IC 48, EC 68	NS

Study	Country / Ethnicity	Tissue source	Control type (IC/EC)	ICH location	Time from ICH onset to biopsy / death	Cases				Controls			
						n	Age in years	% Male	PMI	n	Age in years	% Male	PMI
Tanskanen 2011[26]	Finland / NS	Biopsy and post-mortem	Biopsy and post-mortem (EC & IC)	NS	NS	NS	NS	NS	NS	NS	NS	NS	NS
Vakulenko 1974[27]	Russia / NS	Post-mortem	Contralateral brain tissue (IC)	Supratentorial	NS	22	50-79	50	NS	22	50-79	50	NS
Wang 2011a[28]	China / NS	Biopsy	Distant tissue >1cm from haematoma (IC)	Cases: Lobar 3, deep 25, cerebellar 2. Controls: lobar 1, deep 5, cerebellar 1.	6 patients <6h, 7 patients 6-12h, 5 patients 12-24h, 6 patients 24-72h, 6 patients >72h	30	NS	NS	NA	7	NS	NS	NA
Wang 2011b[29]†	China / NS	Biopsy	Brain tissue from patients undergoing clipping of aneurysm (EC)	Lobar	16 patients 2-6h, 18 patients 7-48h, 13 patients 49-72h	46	59±11*	67	NA	5	56±8*	60	NA
Wang 2004[30]†	China / NS	Post-mortem	Patients who died of causes other than ICH (EC)	NS	<24h	44	59±13*	55	NA	5	NS	NS	NS
Wu 2019[31]†	China / NS	Biopsy	Tissue adjacent to arteriovenous malformation without haemorrhage (EC)	Deep	15±5h*	27	60±8*	59	NA	25	50±9*	56	NA
Wu 2010[32]†	China / NS	Post-mortem	Contralateral brain tissue and brain tissue from patients who died of non-cerebrovascular causes (EC & IC)	Lobar 4, Deep 13, mixed supratentorial 1, mixed infratentorial 4, brainstem 5, unclear 3	43±55 h*	30	58±10*	67	<24h	IC 30, EC 6	IC 58±10, EC 49±15*	IC 67, EC 100	<24h
Wu 2008[33]	China / NS	Post-mortem	Brain tissue from patients who died of non-cerebrovascular causes (EC)	Lobar 4, Deep 13, mixed supratentorial 1, mixed infratentorial 4, brainstem 5, unclear 3	43±55 h*	30	58±10*	67	<24h	EC 6	EC 49±15*	EC 100	<24h
Wu 2006[34]†	China / NS	Cases biopsy, controls post-mortem	“Normal control” brain tissue from coroner’s office (EC)	NS	33h*	29	65**	55	NA	6	NS	NS	<3h
Yilmaz 2009[35]	Germany / NS	Cases Post-mortem, controls not stated	“Healthy tissue” of unspecified origin (EC)	NS	<4 weeks	12	NS	NS	NS	11	NS	NS	NS
Zhang 2019[36]†	China / NS	Biopsy	Distant brain tissue obtained intraoperatively (IC)	Lobar 8, deep 20	7 patients <6h, 7 patients 6-24h, 7 patients 24-72h, 7 patients >72h	28	56±15*	64	NA	28	56±15*	64	NA
Zhang 2015[37]	China / NS	Biopsy	Uncontrolled for ICH status	Deep	6-12h	45	54±11*	64	NA	0	NA	NA	NA

Study	Country / Ethnicity	Tissue source	Control type (IC/EC)	ICH location	Time from ICH onset to biopsy / death	Cases				Controls			
						n	Age in years	% Male	PMI	n	Age in years	% Male	PMI
Zhang 2014[38]†	China / NS	Biopsy	2 patients with intraventricular cyst of unclear aetiology, 5 patients with obstructive hydrocephalus of unclear aetiology, 1 patient with intraventricular meningioma	NS	8 patients <6h, 14 patients 7-12h, 12 patients 13-24h, 8 patients 25-48h, 6h 49-96h, 5h >96h	53	57**	62	NA	8	54**	63	NA
Zhang 2010a[39]†	China / NS	Biopsy	Distant brain tissue from patients undergoing ICH surgery <6h from onset (IC)	NS	10 patients <6h, 8 patients 6-12h, 7 patients 12-24h	25	52±15*	63	NA	5	60±13	NS	NA
Zhang 2010b[40]†	China / NS	Post-mortem	Brain tissue from patients with “other disease without cerebral ischaemia” (EC)	Lobar 4, deep 13, mixed supratentorial 1, brainstem 5, mixed infratentorial 4, unclear 3	43±55h	30	58±10*	66	NS	6	48.5±15 *	100	NS
Zhang 2003[41]	China / NS	Post-mortem	Brain tissue from patients without history of neurological disease (EC)	Lobar 1, deep 6, cerebellar 1	1 patient <24h, 3 patients 24-72h, 4 patients>72h	8	64±13*	63	0.5-2d	5	68±10*	NS	0.5-2d
Zhang 2000[42]	China / NS	Post-mortem	Brain tissue from patients who died of non-neurological disease (EC)	Deep	NS	7	70**	57	9-48h	6	69**	50	NS
Zhao 2018[43]	Case series	Biopsy	Uncontrolled for ICH status	NS	1-7 days	103	52±15*	NS	NA	0	NA	NA	NA
Zhu 2004[44]	China / NS	Biopsy	Biopsy from “tissues in proximity to malformed cerebral veins” (EC)	NS	15 patients <6h, 12 patients 6-48h, 10 patients >48h	37	55**	46	NA	9	NS	NS	NA

Loan JJM, *et al. J Neurol Neurosurg Psychiatry* 2022; 93:126–132. doi: 10.1136/jnnp-2021-327098

Supplementary table 6: Summary of findings.

† included in final meta-analysis dataset; TUNEL – terminal deoxynucleotidyl transferase dUTP nick end labelling; ICH – intracerebral haemorrhage; IHC – immunohistochemistry; WB – western blot; RT-PCR – reverse transcription polymerase chain reaction; RT-qPCR – reverse transcription quantitative polymerase chain reaction; ELISA – enzyme-linked immunosorbent assay; mRNA – messenger ribonucleic acid; EM – electron microscopy; CAA – cerebral amyloid angiopathy

Study	Design	Tissue source	Control type	Analysis method	Molecules studied	Narrative summary of relevant findings
Bao 2011a[1]†	Self-controlled case-control	Biopsy	Distant tissue from unclear hemisphere	IHC, WB, RT-PCR	TUNEL, tropomyosin receptor kinase A, pro-nerve growth factor, neurotrophin receptor p75, sortilin	Increased staining of TUNEL and all molecules except for tropomyosin receptor kinase A in ICH tissue. Neurotrophin receptor P75 and TUNEL staining were correlated
Bao 2011b[2]†	Self-controlled case-control	Biopsy	Tissue from middle cerebral gyrus of unclear hemisphere	IHC	TUNEL, pro-nerve growth factor, neurotrophin receptor p75, sortilin	Increased staining of TUNEL and all molecules except for pro-nerve growth factor in ICH tissue.
Camacho 2019[3]	Case control	Post-mortem	Patients with cerebral amyloid angiopathy but no ICH	IHC, RT-PCR	Apolipoproteins E and J.	Apolipoprotein E was increased in capillaries, meningeal and cortical arteries. Apolipoprotein J was measured in the parenchyma only and was not increased.
Carmichael 2008[4]	Case control	Biopsy	Non-anatomically matched post-mortem “healthy aged” brain tissue.	Microarray	Various mRNA transcripts	624 differentially expressed genes after ICH. Proinflammatory and anti-inflammatory annotated gene networks upregulated.
Chen 2008[5]†	Self-controlled case-control	Biopsy	Distant tissue from unclear hemisphere	IHC , RT-PCR	TUNEL, tumour necrosis factor α (IHC, RT-PCR), BCL2 Associated X Protein (IHC, RT-PCR), BCL2 Like 1 (IHC, RT-PCR), interleukin 1β (RT-PCR), interleukin 6 (RT-PCR)	Tissue appeared most damaged at 24-72h post-ICH. All other molecular markers increased by 72h
Dahnovici 2011[6]	Self-controlled case-control	Post-mortem	Contralateral tissue	IHC	CD68	Increased parenchymal CD68 positive cells close to ICH.
Delgado 2008[7]	Case control	Post-mortem	Contralateral tissue; and tissue from patients with no ICH, inflammatory, or neurological disease	IHC, WB	FAS receptor, FAS ligand	Increased FAS receptor and FAS ligand close to ICH.
Di Napoli 2012[8]	Case control	Post-mortem	Contralateral brain tissue and tissue from patients with no brain pathology	IHC	C reactive protein	Increased C reactive protein staining in neurons, astrocytes and neuropil close to ICH
Duan 2007[9]	Self-controlled case-control	Biopsy	Contralateral brain tissue	IHC	Haem oxygenase 1, B-cell lymphoma 2 protein	Increased haem oxygenase 1 with peak at 17-30h after ICH. Increased B-cell lymphoma 2 protein with peak at 36-96h.
Gang 2018[10]	Self-controlled case-control	Biopsy	Brain tissue >1cm distant from ICH	IHC, WB, RT-qPCR, transmission EM	TUNEL, tumour necrosis factor α, caspase 3, toll-like receptor 4, Myeloid differentiation primary response protein 88, nuclear factor kappa-light-chain-enhancer of activated B cells	Features of apoptosis at <6h with electron microscopy. All studied molecules were upregulated and peak expression within 72h

Study	Design	Tissue source	Control type	Analysis method	Molecules studied	Narrative summary of relevant findings
Guo 2008[11]†	Self-controlled case-control	Biopsy	Normal appearing tissue taken on surgical approach to haematoma	RT-PCR	Aquaporin 4	Increased aquaporin 4 with peak at 12-24h
Hernandez-Guillamon 2012[12]	Case control	Post-mortem	Contralateral brain tissue and tissue from patients who died of cardiorenal failure, gastrointestinal haemorrhage or legionellosis	14C-Benzylamine breakdown assay, IHC, WB	Amine oxidase, copper containing 3, semicarbazide-sensitive amine oxidases	Reduced amine oxidase expression and activity close to ICH. 4 ICH cases diagnosed with CAA
Holfelder 2011[13]	Case control	Post-mortem	Distant tissue from unclear location and tissue from “corresponding locations” of “unaffected brains”	IHC	CD163	Increased parenchymal CD163 stained cells close to ICH. No change in perivascular CD163 staining
Itoh 1997[14]	Case series	Post-mortem	Uncontrolled	IHC	Cistatin C and vascular amyloid costaining	66% of all cases had cystatin C and vascular amyloid costaining. 11 cases diagnosed with CAA had 91% costaining, whilst those without CAA 0% showed costaining.
Jin, 2011[15]	Case control	Cases – biopsy; controls – NS	Unclear origin of control tissue	Fluorescence IHC, confocal microscopy	Neuroglobin	Increased neuroglobin which was colocalised with neuronal nuclear antigen but not glial fibrillary acidic protein
Ke 2007[16]	Case series	Biopsy	Uncontrolled	IHC	Matrix metalloproteinases 2 and 9.	Detected markers in all tissues studied.
Li, 2010[17]	Self-controlled case-control	Post-mortem	Contralateral and distant ipsilateral brain tissue	IHC	Glial fibrillary acidic protein, non-phosphorylated neurofillament	Histological features of apoptosis from 6h of ICH, less prominent in distant tissue. Increased glial fibrillary acidic protein and abnormal neurofilament close to ICH.
Liu 2015[18]	Self-controlled case-control	Biopsy	Distant tissue >1cm from haematoma	Transmission EM, IHC, WB RT-PCR	TUNEL, tumour necrosis factor α, interleukin 1, interleukin 10, haem oxygenase 1, CD163	Increased staining of TUNEL increased abundance of all markers. Interleukin 10 peak at 6-24h then reduced staining relative to control from 24h to >72h trough. Neuronal necrosis and loss of organelles, partly recovered at >72h.
Liu 2006[19]	Case series	Biopsy	Uncontrolled	IHC	TUNEL, hypoxia inducible factor 1 α	For both markers, the number of positive cells increased with time from ICH.
Mantle 2001[20]†	Case control	Biopsy	Patient surgically treated for aneurysm or tumour	Eponymous undescribed techniques. GSH 420 colorimetric assay. WB	Tissue protein glutathione peroxidase, glutathione reductase, catalase, superoxide dismutase and total antioxidant activity. Total glutathione and tissue protein carbonyl.	None of the measured values in the ICH group were significantly different from control
McCarron 1997[21]	Case control	Post-mortem	Tissue from patients with no neuropathologic diagnosis	IHC	Apolipoprotein E, cystatin C, human leucocyte antigen	Positive staining for all markers was more prevalent in the ICH cohort than controls.
Rosell 2011[22]†	Self-controlled case-control	Post-mortem	Contralateral brain tissue	Microarray, RT-qPCR, ELISA	Various mRNA transcripts. Interleukin 8 protein	468 differentially expressed genes in perihaematomal tissue. Up regulated probes were associated with cytokines, chemokines, coagulation factors, cell growth and proliferation. Down regulated probes were associated with cell cycle and neurotrophins.

Study	Design	Tissue source	Control type	Analysis method	Molecules studied	Narrative summary of relevant findings
Rosell 2006[23]	Case control	Post-mortem	Contralateral brain tissue and tissue from patients who died of non-inflammatory disease	IHC, in situ and gelatin zymography	Pro-matrix metalloproteinases 2 and 9 and non-specific gelatinase	Increased pro-matrix metalloproteinase and gelatinase activity particularly in glial cells. No significant change in pro-matrix metalloproteinase 2
Shen 2008[24]	Case series	Biopsy	Uncontrolled	IHC	Glial fibrillary acidic protein, Ki-67, Minichromosome Maintenance Complex Component 2, proliferating cell nuclear antigen, cleaved caspase 3, doublecortin, β tubulin, dihydropyrimidinase like 3, musashi-1, nestin, CD11b, amacrophage/granulocyte antigen	All markers present in ICH tissue, but not quantifiable.
Shtaya 2019[25]	Case control	Post-mortem	Brain tissue distant to the ICH and "Healthy control" brain tissue.	IHC	Ionised calcium binding adaptor molecule 1, CD3	Ionised calcium binding adaptor molecule 1 staining was increased at all time points after ICH compared with control, peaking at 5-12 days after ICH. Stained cells were of transitional, reactive, ameboid and giant morphologies in ICH cases. Parenchymal CD3 positive cells were detectible in 3/27 cases but not in 16-18 controls.
Tanskanen 2011[26]	Case control	Biopsy and post-mortem	Biopsy and post-mortem	IHC	Matrix metalloproteinases 1, 2 7, 9, 19 and 26	Matrix metalloproteinase 2 present in ICH cases, whilst matrix metalloproteinase 1, 7 and 9 were not. Metalloproteinase 19 but not 26 was increased in ICH cases compared with control.
Vakulenko 1974[27]	Self-controlled case-control	Post-mortem	Contralateral brain tissue	Lipid fractionation, Schultz, Okamoto and Fagin testing	Cholestyramine, phospholipid, lecithin, sphingomyelin	Increased cholestyramine in ICH. All lipid fractions reduced in ICH.
Wang 2011a[28]	Self-controlled case-control	Biopsy	Distant tissue >1cm from haematoma	IHC, RT-PCR	TUNEL, redox factor-1, BCL2 Associated X Apoptosis Regulator	Redox factor 1 reduced protein and mRNA maximally 24-72h after ICH. BCL2 Like 1 protein and mRNA increased maximally at 24-72h after ICH. TUNEL increased maximally at 24-72h
Wang 2011b[29]†	Case control	Biopsy	Brain tissue from patients undergoing clipping of aneurysm	IHC	TUNEL, nuclear factor kappa-light-chain-enhancer of activated B cells, interleukin 1b, Intercellular Adhesion Molecule 1	All molecules increased in tissue from cases with ICH
Wang 2004[30]†	Self-controlled case-control	Post-mortem	Patients who died of other causes than ICH	IHC	Glial fibrillary acidic protein, cyclin D1	All molecules increased in tissue from cases with ICH
Wu 2019[31]†	Case control	Biopsy	Tissue adjacent to arteriovenous malformation without haemorrhage	IHC, congo red staining, EM	Light chain 3, beclin-1, cathepsin D	EM demonstrated increased autophagic vesicles in ICH tissue and organelle loss. Staining of all markers was increased in ICH.
Wu 2010[32]†	Case control	Post-mortem	Contralateral brain tissue and brain tissue from patients who died of non-cerebrovascular causes	IHC	Matrix metalloproteinase 9, nuclear factor kappa-light-chain-enhancer of activated B cells, chemokine C-X-C motif ligand 2	Increased staining bilaterally for all markers in ICH tissue. More staining in perihaematomal tissue than contralateral tissue

Loan JJM, *et al. J Neurol Neurosurg Psychiatry* 2022; 93:126–132. doi: 10.1136/jnnp-2021-327098

Study	Design	Tissue source	Control type	Analysis method	Molecules studied	Narrative summary of relevant findings
Wu 2008[33]	Case control	Post-mortem	Brain tissue from patients who died of non-cerebrovascular causes	IHC, in situ RNA hybridisation	Serpin Family E Member 2 (protease nexin 1), thrombin, aquaporin 4	Increased expression of serpin family E member 2. No change in expression of thrombin or aquaporin 4 by immunohistochemistry. Unclear results by in situ RNA hybridisation.
Wu 2006[34]†	Case control	Cases biopsy, controls post-mortem	"Normal control" brain tissue from coroner's office	IHC	B-cell lymphoma 2 protein, BCL2 Associated X Apoptosis Regulator, P53, caspase 3	Increased staining of all markers
Yilmaz 2009[35]	Case control	Cases Post-mortem, controls not stated	"healthy tissue" of unspecified origin	IHC	CD209, CD123, CD3, Human leukocyte antigen-DR	Increased staining of all markers measured
Zhang 2019[36]†	Self-controlled case-control	Biopsy	Distant brain tissue obtained intraoperatively	IHC, RT-qPCR, WB	tumour necrosis factor α, heme-oxygenase 1, interleukin 1β	All markers increased from 6h measured by IHC and RT-qPCR. All markers increased at all time points by WB.
Zhang 2015[37]	Case series	Biopsy	Uncontrolled for ICH status	IHC	Nuclear factor kappa-light-chain-enhancer of activated B cells, glial fibrillary acidic protein, neuron-specific enolase	Greater staining of nuclear factor kappa-light-chain-enhancer of activated B cells associated with poor outcome at 6 months post-ICH. Nuclear factor kappa-light-chain-enhancer of activated B cells costained with glial fibrillary acidic protein and neuron-specific enolase.
Zhang 2014[38]†	Case control	Biopsy	2 patients with intraventricular cyst of unclear aetiology, 5 patients with obstructive hydrocephalus of unclear aetiology, 1 patient with intraventricular meningioma	IHC	TUNEL, nuclear factor kappa-light-chain-enhancer of activated B cells, glial fibrillary acidic protein, interleukin 1β, tumour necrosis factor α	Increased staining of all markers in ICH tissue
Zhang 2010a[39]†	Self-controlled case-control	Biopsy	Distant brain tissue from patients undergoing ICH surgery <6h from onset	IHC	TUNEL, matrix metalloproteinase, caspase 3	All markers increased at all time points. Most induced in the 6-12 and 12-24h groups
Zhang 2010b[40]†	Case control	Post-mortem	Brain tissue from patients with "other disease without cerebral ischaemia"	IHC, in situ RNA hybridisation	Thrombin, Serpin Family E Member 2, Coagulation Factor II Thrombin Receptor	Increased Serpin Family E Member 2 by in situ RNA hybridisation but not IHC. Increased thrombin and Coagulation Factor II Thrombin Receptor in ICH tissue.
Zhang 2003[41]	Case control	Post-mortem	Brain tissue from patients without history of neurological disease	IHC	Glial fibrillary acidic protein, endothelin 1	Both markers increased in ICH tissue
Zhang 2000[42]	Case control	Post-mortem	Patients who died of non-neurological disease	IHC	Glucose transporter 1	Increased Glut-1 staining in endothelial cells peaked at 24h. Peaked at 72h in astrocytes
Zhao 2018[43]	Case series	Biopsy	Uncontrolled for ICH status	IHC	Nuclear factor kappa-light-chain-enhancer of activated B cells	Staining appeared "increased" on days 1, 3 and 7. Peak on day 3
Zhu 2004[44]	Case control	Biopsy	Biopsy from "tissues in proximity to malformed cerebral veins"	IHC	TUNEL, hypoxia inducible factor 1α	All markers increased staining in ICH tissue

Supplementary table 7: Risk of bias.

Green shading indicates criteria where points were assigned

Study	Is case definition adequate?	Representativeness of the cases	Control population	Haemorrhage status of controls	Comparability of cases and controls on the basis of design or analysis	Tissues processing and batch effects	Same method of case ascertainment for cases and controls	Were all cases and controls accounted for in all analyses?	Score: Max 9
Bao 2011a[1]	Yes: independent validation	Potential for selection biases	Tissue from same person as case but distant to ICH	Distant brain tissue	Controls for between-subjects effects but no anatomical matching	Same processing technique, batch effect possible	Yes	Data missing	4
Bao 2011b[2]	Yes: independent validation	Potential for selection biases	Tissue from same person as case but distant to ICH	Distant brain tissue	Controls for between-subjects effects but no anatomical matching	Same processing technique, batch effect possible	Yes	Yes	5
Camacho 2019[3]	Yes: independent validation	Potential for selection biases	Hospital controls	Patients with no history of ICH	Controls for presence of cerebral amyloid angiopathy	Same processing technique, batch effect possible	Yes	Yes	5
Carmichael 2008[4]	Yes: independent validation	Potential for selection biases	Unclear population of origin	Patients with no history of ICH	Age and anatomical matching	Different platforms. No batch effect identified	No	Data missing but explanation provided	3
Chen 2008[5]	Yes: independent validation	Potential for selection biases	Tissue from same person as case but distant to ICH	Distant brain tissue	Controls for between-subjects effects but no anatomical matching	Same processing technique, batch effect possible	Unclear	Yes	4
Dahnovici 2011[6]	Not described	Potential for selection biases	Tissue from same person as case but distant to ICH	Distant brain tissue	Controls for between-subjects effects. Anatomical matching	Same processing technique, batch effect possible	Unclear	Unclear	3
Delgado 2008[7]	Yes: independent validation	Potential for selection biases	Tissue from same person as case but distant to ICH and hospital controls	Patients with no history of ICH and distant brain tissue from patients with ICH	Controls for between subjects effects, anatomical matching and distant effects of ICH	All tissues processed in the same manner with equal numbers of cases and controls per batch	Unclear for non-ICH controls	Unclear	6

Loan JJM, *et al. J Neurol Neurosurg Psychiatry* 2022; 93:126–132. doi: 10.1136/jnnp-2021-327098

Study	Is case definition adequate?	Representativeness of the cases	Control population	Haemorrhage status of controls	Comparability of cases and controls on the basis of design or analysis	Tissues processing and batch effects	Same method of case ascertainment for cases and controls	Were all cases and controls accounted for in all analyses?	Score: Max 9
Di Napoli 2012[8]	Yes: independent validation	Potential for selection biases	Tissue from same person as case but distant to ICH and hospital controls	Patients with no history of ICH and distant brain tissue from patients with ICH	No data on selection of control cases, cases with bilateral sampling or anatomical location	Same processing technique, batch effect possible	Unclear for non-ICH controls	Yes	4
Duan 2007[9]	Yes: independent validation	Potential for selection biases	Tissue from same person as case but distant to ICH	Distant brain tissue	Controls for between subjects effects,	Same processing technique, batch effect possible	Yes	Yes	5
Gang 2018[10]	Yes: independent validation	Potential for selection biases	Tissue from same person as case but distant to ICH	Distant brain tissue	Controls for between-subjects effects but no anatomical matching	All tissues processed in the same manner with equal numbers of cases and controls per batch	Yes	Unclear	5
Guo 2008[11]	Yes: independent validation	Potential for selection biases	Tissue from same person as case but distant to ICH	Distant brain tissue	Controls for between-subjects effects but no anatomical matching	Same processing technique, batch effect possible	Unclear	Yes	4
Hernandez-Guillamon 2012[12]	Yes: independent validation	Potential for selection biases	Tissue from same person as case but distant to ICH and hospital controls	Patients with no history of ICH and distant brain tissue from patients with ICH	Controls for between-subjects effects but no anatomical matching	Same processing technique, batch effect possible	Unclear for non-ICH controls	Yes	5
Holfelder 2011[13]	"Intracerebral bleeding" not defined	Potential for selection biases	Tissue from same person as case but distant to ICH and hospital controls	Patients with no history of ICH and distant brain tissue from patients with ICH	Unmatched for age, some anatomical matching	Same processing technique, batch effect possible	Unclear for non-ICH controls	Data missing but explanation provided	3
Jin, 2011[15]	Yes: independent validation	Potential for selection biases	Not described	Not described	Not described	Same processing technique, batch effect possible	Not described	Unclear	1
Li, 2010[17]	Yes: independent validation	Consecutive autopsy series	Tissue from same person as case but distant to ICH	Distant brain tissue	Controls for between-subjects effects. Anatomical matching	Yes	Yes	Unclear	7

Study	Is case definition adequate?	Representativeness of the cases	Control population	Haemorrhage status of controls	Comparability of cases and controls on the basis of design or analysis	Tissues processing and batch effects	Same method of case ascertainment for cases and controls	Were all cases and controls accounted for in all analyses?	Score: Max 9
Liu 2015[18]	Yes: independent validation	Potential for selection biases	Tissue from same person as case but distant to ICH	Distant brain tissue	Controls for between-subjects effects but no anatomical matching	Same processing technique, batch effect possible	No	Yes	4
Mantle 2001[20]	No description	Potential for selection biases	Hospital controls	Patients treated for aneurysm or undergoing tumour surgery.	No matching described	Same processing technique, batch effect possible	Unclear	Unclear	0
McCarron 1997[21]	Yes: independent validation	Potential for selection biases	Community controls	Patients with no history of ICH	Age matching, controls for amyloid angiopathy	Same processing technique, batch effect possible	Unclear	Yes	6
Rosell 2011[22]	Yes: independent validation	Potential for selection biases	Tissue from same person as case but distant to ICH	Distant brain tissue	Controls for between-subjects effects. Some anatomical matching	Same processing technique, batch effect possible	Unclear	Yes	5
Rosell 2006[23]	Yes: independent validation	Potential for selection biases	Tissue from same person as case but distant to ICH and community controls	Patients with no history of ICH and distant brain tissue from patients with ICH	Controls for between-subjects effects. Anatomical matching	Same processing technique, batch effect possible	Yes	Yes	7
Shtaya 2019[25]	Yes: independent validation	Potential for selection biases	Tissue from same person as case but distant to ICH and hospital controls	Patients with no history of ICH and distant brain tissue from patients with ICH	Controls for between-subjects effects, age and anatomical matching	Same processing technique, batch effect possible	Unclear	Unclear	5
Tanskanen 2011[26]	Yes: independent validation	Potential for selection biases	Hospital controls	Patients with no history of ICH	Control for CAA	Same processing technique, batch effect possible	Unclear	Unclear	3
Vakulenko 1974[27]	Yes: independent validation	Potential for selection biases	Tissue from same person as case but distant to ICH	Distant brain tissue	Controls for between-subjects effects but no anatomical matching	Same processing technique, batch effect possible	Unclear	Yes	4

Study	Is case definition adequate?	Representativeness of the cases	Control population	Haemorrhage status of controls	Comparability of cases and controls on the basis of design or analysis	Tissues processing and batch effects	Same method of case ascertainment for cases and controls	Were all cases and controls accounted for in all analyses?	Score: Max 9
Wang 2011a[28]	Yes: independent validation	Potential for selection biases	Tissue from same person as case but distant to ICH	Distant brain tissue	Controls for between-subjects effects but no anatomical matching	Same processing technique, batch effect possible	No	Yes	4
Wang 2011b[29]	Yes: independent validation	Potential for selection biases	Hospital controls	Patients with no history of ICH	Controls for shared risk factors for ICH and aneurysm formation. Age and sex matched	Same processing technique, batch effect possible	Unclear	Yes	5
Wang 2004[30]	Yes: independent validation	Potential for selection biases	No description	Patients with no history of ICH	None	Same processing technique, batch effect possible	No	Yes	3
Wu 2019[31]	Yes: independent validation	Potential for selection biases	Hospital controls	Patients with no history of ICH	Controls for age and tissue retrieval method	Same processing technique, batch effect possible	Unclear	Yes	5
Wu 2010[32]	No description	Potential for selection biases	Tissue from same person as case but distant to ICH and hospital controls	Patients with no history of ICH and distant brain tissue from patients with ICH	Controls for between-subjects effects and anatomical matching	Same processing technique, batch effect possible	Unclear	Yes	5
Wu 2008[33]	No description	Potential for selection biases	Hospital controls	Patients with no history of ICH	Anatomical matching only	Same processing technique, batch effect possible	Unclear	Unclear	2
Wu 2006[34]	Yes: independent validation	Potential for selection biases	Hospital controls	Patients with no history of ICH	None	Same processing technique, batch effect possible	No	Yes	3
Yilmaz 2009[35]	Yes: independent validation	Potential for selection biases	Healthy tissue of undescribed origin	Patients with no history of ICH	Anatomical matching only	Same processing technique, batch effect possible	Unclear	Yes	4

Loan JJM, *et al. J Neurol Neurosurg Psychiatry* 2022; 93:126–132. doi: 10.1136/jnnp-2021-327098

**Study**	**Is case definition adequate?**	**Representativeness of the cases**	**Control population**	**Haemorrhage status of controls**	**Comparability of cases and controls on the basis of design or analysis**	**Tissues processing and batch effects**	**Same method of case ascertainment for cases and controls**	**Were all cases and controls accounted for in all analyses?**	**Score: Max 9**
Zhang 2019[36]	Yes: independent validation	Potential for selection biases	Tissue from same person as case but distant to ICH	Distant brain tissue	Controls for between-subjects effects but no anatomical matching	Same processing technique, batch effect possible	Yes	Unclear	4
Zhang 2014[38]	Yes: independent validation	Potential for selection biases	Hospital controls	Patients with no history of ICH	Age and sex matched	Same processing technique, batch effect possible	Unclear	Yes	4
Zhang 2010a[39]	Yes: independent validation	Potential for selection biases	Tissue from same person as case but distant to ICH	Distant brain tissue	Controls for between-subjects effects but no anatomical matching	Same processing technique, batch effect possible	Unclear	Yes	4
Zhang 2010b[40]	Yes: independent validation	Potential for selection biases	Hospital controls	Patients with no history of ICH	Anatomical matching	Same processing technique, batch effect possible	Unclear	Yes	4
Zhang 2003[41]	Yes: independent validation	Potential for selection biases	Healthy tissue of undescribed origin	Patients with no history of ICH	Anatomical matching only	Same processing technique, batch effect possible	Unclear	Yes	4
Zhang 2000[42]	Yes: independent validation	Potential for selection biases	Hospital controls	Patients with no history of ICH	None	Same processing technique, batch effect possible	Yes	Yes	4
Zhu 2004[44]	No description	Potential for selection biases	Hospital controls	Unclear	Unclear	Same processing technique, batch effect possible	Unclear	Yes	1

Loan JJM, *et al. J Neurol Neurosurg Psychiatry* 2022; 93:126–132. doi: 10.1136/jnnp-2021-327098

Supplementary figure 1: Forest plot of pooled independent associations of interleukin-1*β* protein with ICH stratified by time from ICH onset to tissue retrieval.

A – tissue retrieved <6h; B – tissue retrieved >6h after ICH onset. Tissue analysed by immunohistochemistry. Studies of surgically resected tissue compared with healthy access tissue that was unaffected by ICH, retrieved at any time point (Zhang 2019[36]) or from controls undergoing surgery for non-haemorrhagic disease (Wang 2011b[29] and Zhang 2014[38]).

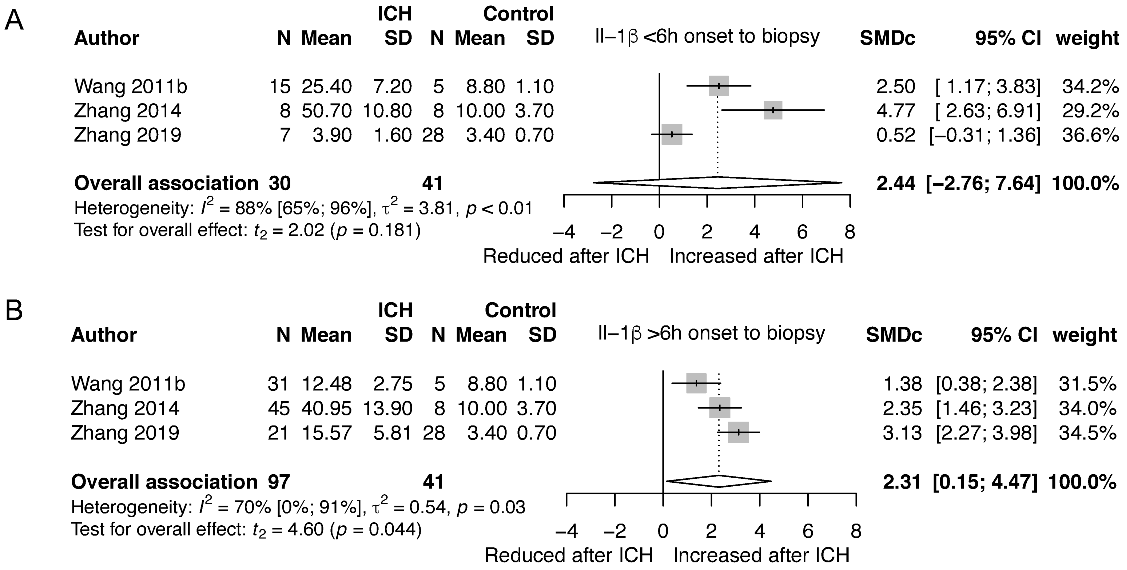

Loan JJM, *et al. J Neurol Neurosurg Psychiatry* 2022; 93:126–132. doi: 10.1136/jnnp-2021-327098

Supplementary figure 2: Funnel plot of studies included in meta-analysis of associations of interleukin-1β with ICH.
The symmetrical distribution indicates a low probability of publication bias.

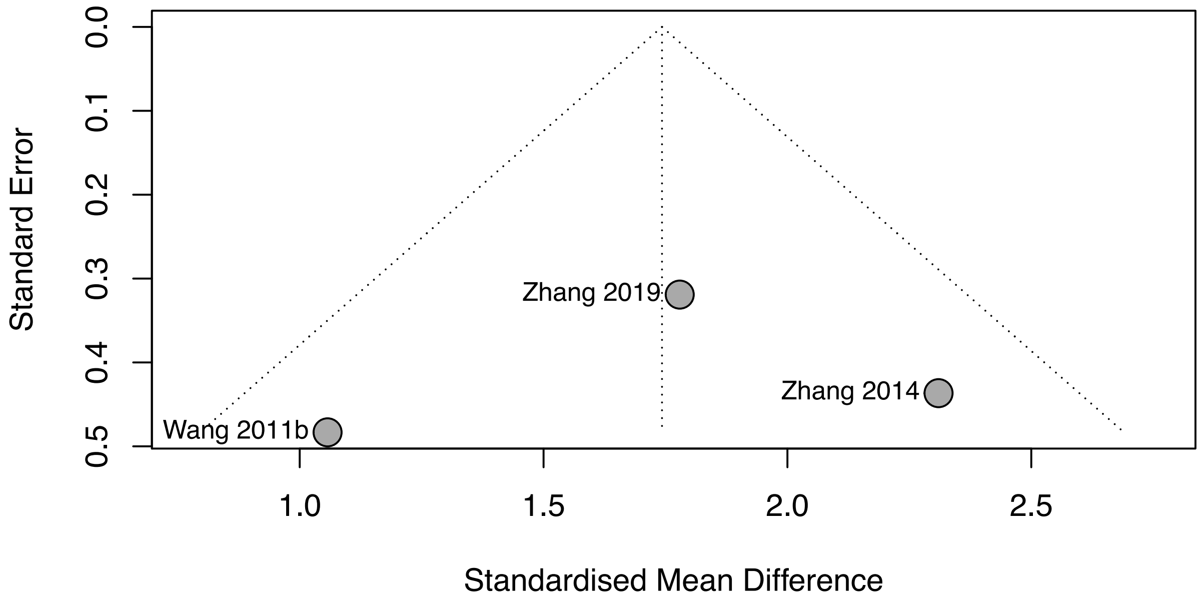

Loan JJM, *et al. J Neurol Neurosurg Psychiatry* 2022; 93:126–132. doi: 10.1136/jnnp-2021-327098

Supplementary figure 3: Gene symbols contributing to enrichment of significantly enriched GO biological process terms.

Gene symbols ranked by number of significantly enriched GO terms that are increased or decreased after ICH. GO term enrichment in sets of genes analysed by one, two or three studies.

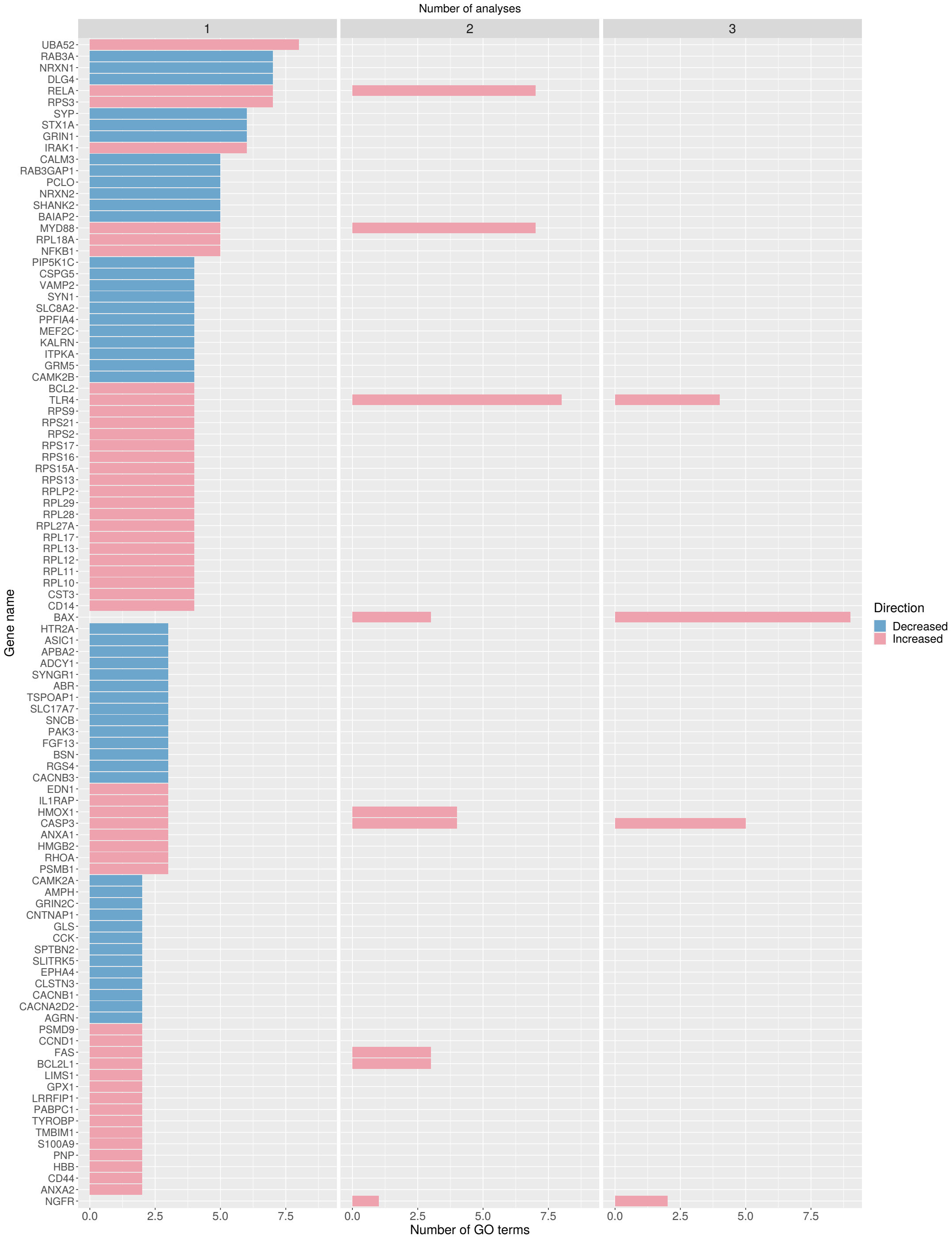

Loan JJM, *et al. J Neurol Neurosurg Psychiatry* 2022; 93:126–132. doi: 10.1136/jnnp-2021-327098

Supplementary figure 4: Chord diagrams of significantly enriched GO terms.
Segments indicate statistically significantly enriched GO terms at each level of replication (Fishers p<0.05). Chords indicate shared gene symbols. Chord width is weighted by study replication with larger chords connecting GO terms that are enriched in more highly replicated gene sets. Large groups of gene symbols drive specific enrichment of several GO terms derived from gene sets that are reduced (panel B) after ICH. No such pattern is seen in GO terms enriched in sets of genes that are increased after ICH (panel A). In this set, gene symbols contributing to enrichment of GO terms overlap considerably.

A

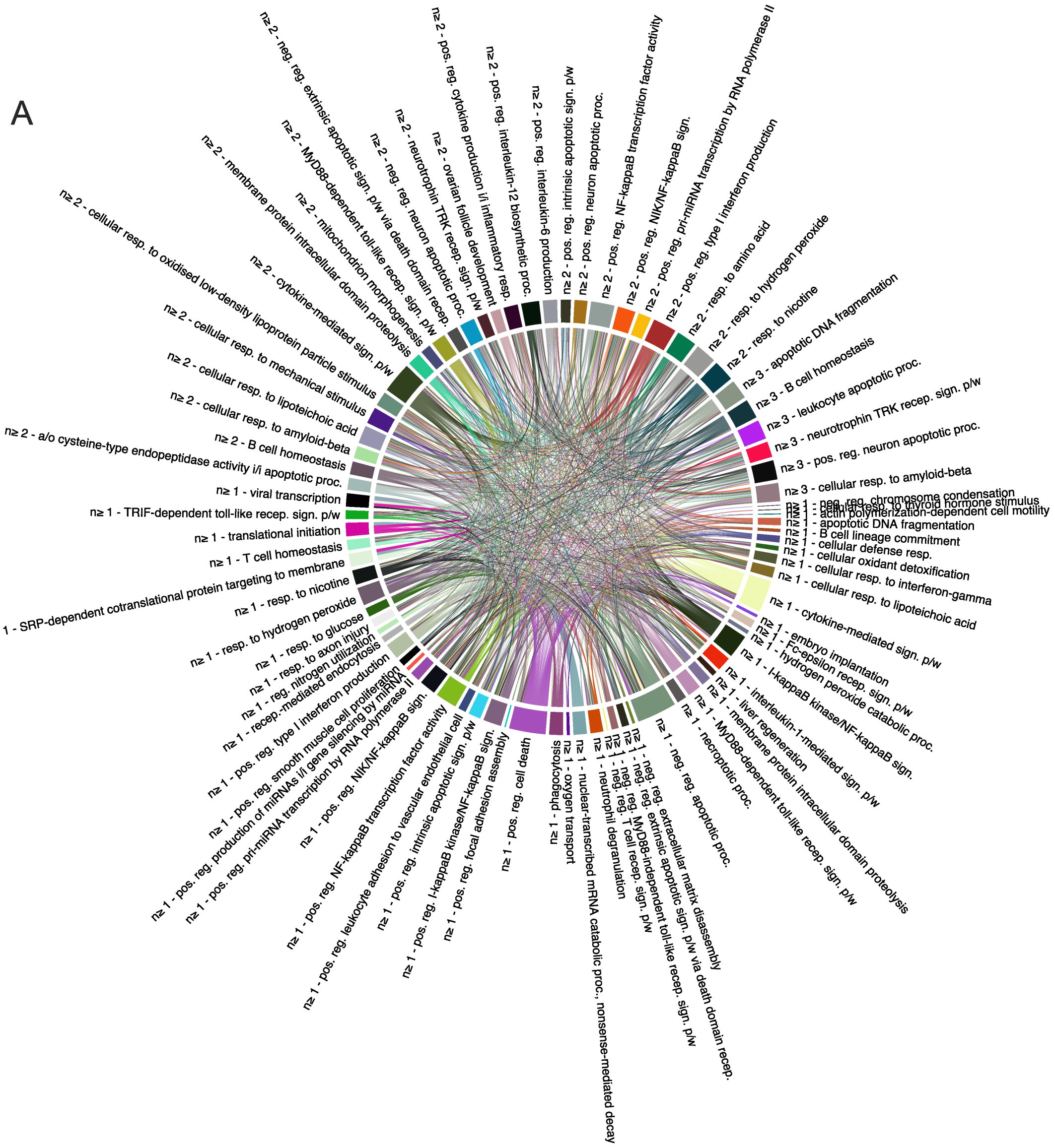

Increased >1.5 fold

B

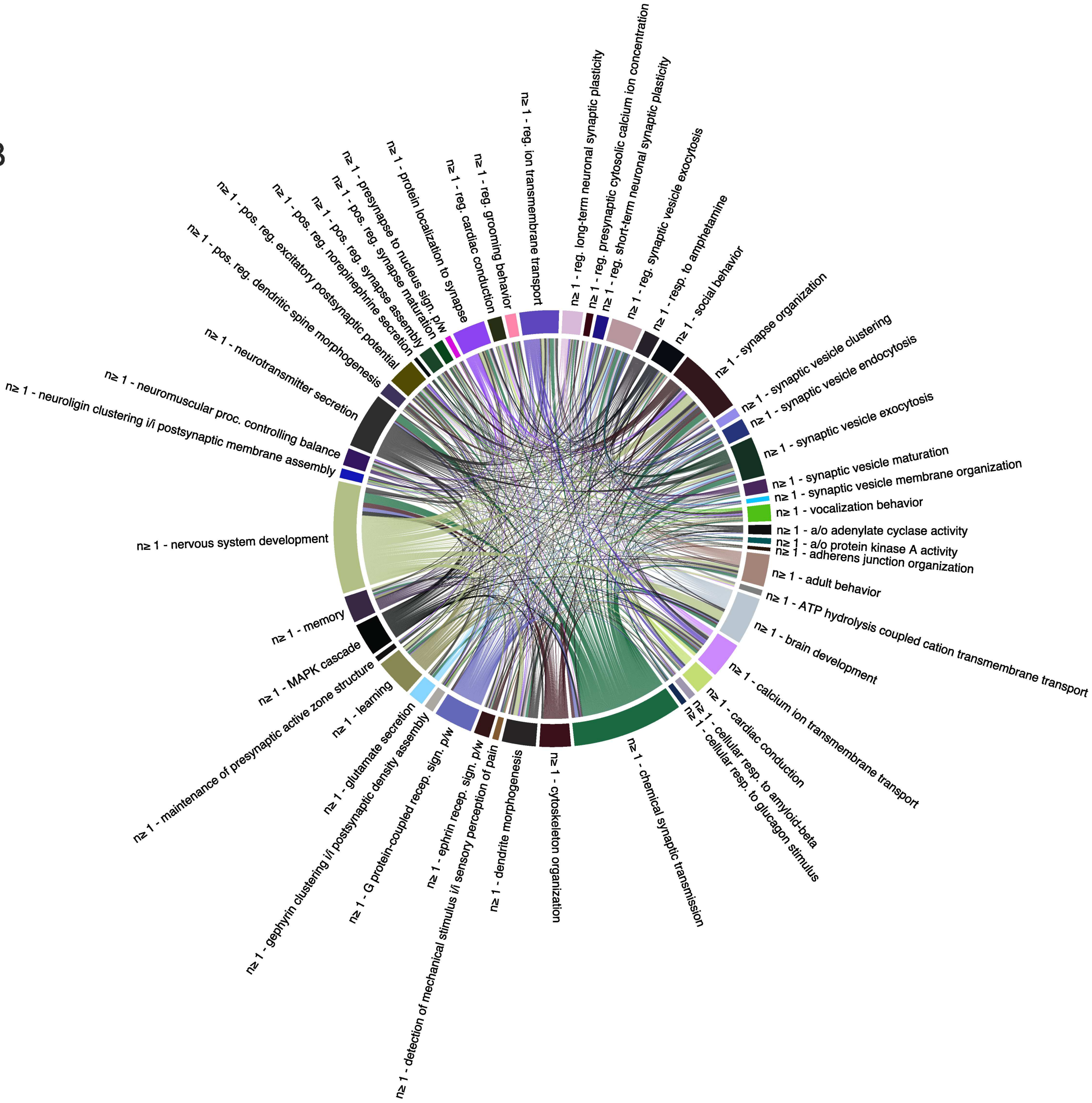

Reduced >1.5 fold

Supplementary table 8: Enrichment table

Enriched Gene Ontology: Biological Process terms in sets of genes that are increased or decreased after ICH. For each set, the 50 terms with the lowest p-value are displayed. Significantly enriched (p<0.05) terms with >1 gene found in the meta-analysis set are tabulated.

GO ID	GO Term	Total annotated genes in background set (n)	Genes in meta-analysis set (n)	p-value (Fishers)
**Gene set: increased in ≥1 study**				
GO:0043312	neutrophil degranulation	326	36	7·20E-19
GO:0006614	SRP-dependent cotranslational protein targeting to membrane	94	19	3·10E-15
GO:0000184	nuclear-transcribed mRNA catabolic process, nonsense-mediated decay	116	20	8·30E-14
GO:0006413	translational initiation	171	21	3·00E-12
GO:0019083	viral transcription	167	19	1·30E-10
GO:0002755	MyD88-dependent toll-like receptor signaling pathway	20	6	9·80E-07
GO:1902042	negative regulation of extrinsic apoptotic signaling pathway via death domain receptors	22	6	1·80E-06
GO:0051092	positive regulation of NF-kappaB transcription factor activity	101	11	1·80E-06
GO:1901224	positive regulation of NIK/NF-kappaB signaling	45	8	1·90E-06
GO:0042542	response to hydrogen peroxide	102	11	2·20E-06
GO:0007566	embryo implantation	32	8	2·30E-06
GO:0019221	cytokine-mediated signaling pathway	425	34	9·30E-06
GO:0048678	response to axon injury	57	7	1·20E-05
GO:0043066	negative regulation of apoptotic process	575	36	1·20E-05
GO:0032481	positive regulation of type I interferon production	59	8	1·80E-05
GO:0070498	interleukin-1-mediated signaling pathway	84	9	1·90E-05
GO:0002326	B cell lineage commitment	4	3	2·30E-05
GO:0097067	cellular response to thyroid hormone stimulus	12	4	4·50E-05
GO:0034128	negative regulation of MyD88-independent toll-like receptor signaling pathway	5	3	5·60E-05
GO:0070358	actin polymerization-dependent cell motility	5	3	5·60E-05
GO:0042744	hydrogen peroxide catabolic process	13	4	6·40E-05
GO:2001244	positive regulation of intrinsic apoptotic signaling pathway	47	9	6·40E-05
GO:0070266	necroptotic process	28	6	6·90E-05
GO:0071346	cellular response to interferon-gamma	96	11	7·40E-05
GO:0010942	positive regulation of cell death	481	29	0·00011
GO:0006968	cellular defense response	15	4	0·00012
GO:0006898	receptor-mediated endocytosis	165	9	0·00013
GO:0006309	apoptotic DNA fragmentation	17	4	0·00019
GO:0015671	oxygen transport	7	3	0·00019
GO:0071223	cellular response to lipoteichoic acid	7	3	0·00019
GO:0006909	phagocytosis	152	12	0·00021
GO:0038095	Fc-epsilon receptor signaling pathway	91	8	0·00022
GO:0031293	membrane protein intracellular domain proteolysis	18	4	0·00025
GO:0007249	I-kappaB kinase/NF-kappaB signaling	183	13	0·00028
GO:1904996	positive regulation of leukocyte adhesion to vascular endothelial cell	8	3	0·0003
GO:0048661	positive regulation of smooth muscle cell proliferation	55	7	0·00031
GO:0010716	negative regulation of extracellular matrix disassembly	2	2	0·00032
GO:1902340	negative regulation of chromosome condensation	2	2	0·00032
GO:0006808	regulation of nitrogen utilization	2	2	0·00032
GO:0035094	response to nicotine	25	6	0·00038
GO:0043029	T cell homeostasis	22	5	0·00038
GO:0097421	liver regeneration	20	4	0·00039
GO:1903800	positive regulation of production of miRNAs involved in gene silencing by miRNA	9	3	0·00044
GO:0043123	positive regulation of I-kappaB kinase/NF-kappaB signaling	129	9	0·00052
GO:0098869	cellular oxidant detoxification	57	6	0·00053
GO:0051894	positive regulation of focal adhesion assembly	22	4	0·00057
GO:1902895	positive regulation of pri-miRNA transcription by RNA polymerase II	22	4	0·00057
GO:0035666	TRIF-dependent toll-like receptor signaling pathway	22	4	0·00057
GO:0009749	response to glucose	137	7	0·0007
GO:0050860	negative regulation of T cell receptor signaling pathway	11	3	0·00085
**Gene set: increased in ≥2 studies**				

GO:1902042	negative regulation of extrinsic apoptotic signaling pathway via death domain receptors	22	3	1·30E-06
GO:0045084	positive regulation of interleukin-12 biosynthetic process	3	2	3·00E-06
GO:0043123	positive regulation of I-kappaB kinase/NF-kappaB signaling	129	4	6·70E-06
GO:0071260	cellular response to mechanical stimulus	54	3	2·10E-05
GO:0071223	cellular response to lipoteichoic acid	7	2	2·10E-05
GO:0140052	cellular response to oxidised low-density lipoprotein particle stimulus	7	2	2·10E-05
GO:1900017	positive regulation of cytokine production involved in inflammatory response	10	2	4·50E-05
GO:0051092	positive regulation of NF-kappaB transcription factor activity	101	3	0·00013
GO:0031293	membrane protein intracellular domain proteolysis	18	2	0·00015
GO:0035094	response to nicotine	25	3	0·00015
GO:0043524	negative regulation of neuron apoptotic process	108	3	0·00016
GO:0001782	B cell homeostasis	19	2	0·00017
GO:0002755	MyD88-dependent toll-like receptor signaling pathway	20	2	0·00019
GO:0070584	mitochondrion morphogenesis	20	2	0·00019
GO:1902895	positive regulation of pri-miRNA transcription by RNA polymerase II	22	2	0·00023
GO:1904646	cellular response to amyloid-beta	25	2	0·00029
GO:0043200	response to amino acid	79	3	0·00031
GO:0048011	neurotrophin TRK receptor signaling pathway	28	2	0·00037
GO:0032481	positive regulation of type I interferon production	59	3	0·0004
GO:0042542	response to hydrogen peroxide	102	3	0·00048
GO:0001541	ovarian follicle development	33	2	0·00052
GO:0032755	positive regulation of interleukin-6 production	40	2	0·00076
GO:0043525	positive regulation of neuron apoptotic process	41	2	0·0008
GO:0019221	cytokine-mediated signaling pathway	425	5	0·00084
GO:0006919	activation of cysteine-type endopeptidase activity involved in apoptotic process	53	3	0·00086
GO:1901224	positive regulation of NIK/NF-kappaB signaling	45	2	0·00096
GO:2001244	positive regulation of intrinsic apoptotic signaling pathway	47	2	0·00105
**Gene set: increased in ≥3 studies**				
GO:0001782	B cell homeostasis	19	2	3·10E-05
GO:1904646	cellular response to amyloid-beta	25	2	5·40E-05
GO:0048011	neurotrophin TRK receptor signaling pathway	28	2	6·80E-05
GO:0043525	positive regulation of neuron apoptotic process	41	2	0·00015
GO:0071887	leukocyte apoptotic process	60	2	0·00153
**Gene set: decreased in ≥1 study**				
GO:2000463	positive regulation of excitatory postsynaptic potential	25	7	1·50E-06
GO:0014047	glutamate secretion	35	9	1·90E-06
GO:0048172	regulation of short-term neuronal synaptic plasticity	12	5	5·50E-06
GO:0048169	regulation of long-term neuronal synaptic plasticity	24	6	1·70E-05
GO:0035418	protein localization to synapse	51	12	1·90E-05
GO:0050885	neuromuscular process controlling balance	36	7	2·00E-05
GO:0070588	calcium ion transmembrane transport	194	19	5·50E-05
GO:0007269	neurotransmitter secretion	124	23	8·70E-05
GO:0007268	chemical synaptic transmission	467	57	8·80E-05
GO:0061003	positive regulation of dendritic spine morphogenesis	20	5	9·10E-05
GO:0099509	regulation of presynaptic cytosolic calcium ion concentration	11	4	9·60E-05
GO:2000300	regulation of synaptic vesicle exocytosis	59	11	9·70E-05
GO:0050808	synapse organization	328	31	0·0001
GO:0061337	cardiac conduction	96	13	0·00017
GO:0035176	social behavior	35	6	0·00017
GO:0016188	synaptic vesicle maturation	7	4	0·00026
GO:0071625	vocalization behavior	14	4	0·00028
GO:0007399	nervous system development	1653	83	0·00034
GO:0097091	synaptic vesicle clustering	16	4	0·00048
GO:0007420	brain development	522	30	0·00054
GO:0099526	presynapse to nucleus signaling pathway	2	2	0·00058
GO:0010701	positive regulation of norepinephrine secretion	2	2	0·00058
GO:1903779	regulation of cardiac conduction	52	7	0·00068
GO:0007612	learning	120	14	0·00068
GO:0090129	positive regulation of synapse maturation	8	3	0·00071
GO:0007613	memory	91	10	0·00073
GO:0048813	dendrite morphogenesis	121	14	0·00095
GO:0048488	synaptic vesicle endocytosis	35	7	0·00114

GO:0001975	response to amphetamine	20	4	0·00119
GO:0007190	activation of adenylate cyclase activity	20	4	0·00119
GO:0030534	adult behavior	96	10	0·00133
GO:0050966	detection of mechanical stimulus involved in sensory perception of pain	10	3	0·00147
GO:0099132	ATP hydrolysis coupled cation transmembrane transport	42	5	0·00171
GO:2000821	regulation of grooming behavior	3	2	0·00172
GO:0097116	gephyrin clustering involved in postsynaptic density assembly	3	2	0·00172
GO:0048499	synaptic vesicle membrane organization	3	2	0·00172
GO:0000165	MAPK cascade	564	25	0·00179
GO:0051965	positive regulation of synapse assembly	55	7	0·0018
GO:0007186	G protein-coupled receptor signaling pathway	426	29	0·00197
GO:0048013	ephrin receptor signaling pathway	74	7	0·00201
GO:0007010	cytoskeleton organization	815	30	0·00222
GO:0016079	synaptic vesicle exocytosis	87	16	0·00229
GO:0034765	regulation of ion transmembrane transport	300	23	0·0025
GO:1904646	cellular response to amyloid-beta	25	4	0·00282
GO:0070327	thyroid hormone transport	4	2	0·00338
GO:0097118	neuroligin clustering involved in postsynaptic membrane assembly	4	2	0·00338
GO:0048790	maintenance of presynaptic active zone structure	4	2	0·00338
GO:0034332	adherens junction organization	113	7	0·00426
GO:0034199	activation of protein kinase A activity	15	3	0·00511
GO:0071377	cellular response to glucagon stimulus	15	3	0·00511

Loan JJM, *et al. J Neurol Neurosurg Psychiatry* 2022; 93:126–132. doi: 10.1136/jnnp-2021-327098

References for supplementary materials

1 Bao G, Han Y, Wang M, *et al.* Relationship between cellular apoptosis and the expression of p75 neurotrophin receptor and tyrosine kinase A receptor in tissue surrounding haematoma in intracerebral haemorrhage. *J Int Med Res* 2011;**39**:150–60. doi:10.1177/147323001103900116

2 Bao G, Li Q, Han Y, *et al.* Role of the nerve growth factor precursor-neurotrophin receptor p75 and sortilin pathway on apoptosis in the brain of patients with intracerebral hemorrhage. *Neural Regeneration Research* 2011;**6**:1696–700.

3 Camacho J, Moliné T, Bonaterra-Pastra A, *et al.* Brain ApoA-I, ApoJ and ApoE Immunodetection in Cerebral Amyloid Angiopathy. *Front Neurol* 2019;**10**:187. doi:10.3389/fneur.2019.00187

4 Carmichael ST, Vespa PM, Saver JL, *et al.* Genomic profiles of damage and protection in human intracerebral hemorrhage. *J Cereb Blood Flow Metab* 2008;**28**:1860–75. doi:10.1038/jcbfm.2008.77

5 Chen L, Guo F, Li X, *et al.* Real-time analysis of inflammatory cytokines and regulatory gene expression in tissues surrounding the hematoma after intracerebral hemorrhage. *Neural Regeneration Research* 2008;**3**:1197–202.

6 Dahnovici RM, Pintea IL, Mălăescu DG, *et al.* Microscopic aspects of macrophage system cells in hemorrhagic stroke in humans. *Rom J Morphol Embryol* 2011;**52**:1249–53.

7 Delgado P, Cuadrado E, Rosell A, *et al.* Fas system activation in perihematomal areas after spontaneous intracerebral hemorrhage. *Stroke* 2008;**39**:1730–4. doi:10.1161/STROKEAHA.107.500876

8 Di Napoli M, Godoy DA, Campi V, *et al.* C-reactive protein in intracerebral hemorrhage: time course, tissue localization, and prognosis. *Neurology* 2012;**79**:690–9. doi:10.1212/WNL.0b013e318264e3be

9 Duan S, Wang X, Wang C, *et al.* [Expressions of heme oxygenase-1 and apoptosis-modulating proteins in peri-hematoma cortex after intracerebral hemorrhage in human being]. *Zhonghua Yi Xue Za Zhi* 2007;**87**:1904–7.

10 Gang X, Han Q, Zhao X, *et al.* Dynamic Changes in Toll-Like Receptor 4 in Human Perihematoma Tissue after Intracerebral Hemorrhage. *World Neurosurg* 2018;**118**:e593–600. doi:10.1016/j.wneu.2018.06.247

11 Guo F, Xu Y, Chen L, *et al.* [The relationship between the aquaporin-4 and brain edema, pathologic change, ultrastructure in peri-hematoma tissue in patients with intracerebral hemorrhage]. *Zhongguo Wei Zhong Bing Ji Jiu Yi Xue* 2008;**20**:674–7.

12 Hernandez-Guillamon M, Solé M, Delgado P, *et al.* VAP-1/SSAO plasma activity and brain expression in human hemorrhagic stroke. *Cerebrovasc Dis* 2012;**33**:55–63. doi:10.1159/000333370

13 Holfelder K, Schittenhelm J, Trautmann K, *et al.* De novo expression of the hemoglobin scavenger receptor CD163 by activated microglia is not associated with hemorrhages in human brain lesions. *Histol Histopathol* 2011;**26**:1007–17. doi:10.14670/HH-26.1007

14 Itoh Y, Yamada M. Cerebral amyloid angiopathy in the elderly: the clinicopathological features, pathogenesis, and risk factors. *J Med Dent Sci* 1997;**44**:11–9.

15 Jin K, Mao X, Xie L, *et al.* Neuroglobin expression in human arteriovenous malformation and intracerebral hemorrhage. *Acta Neurochir Suppl* 2011;**111**:315–9. doi:10.1007/978-3-7091-0693-8_52

16 Ke S, Jin X, Zhang K, *et al.* [Study on expression of matrix metalloproteinase-2 and matrix metalloproteinase-9 in brain tissue adjacent to hemorrhage after brain hemorrhage in human]. *Zhongguo Wei Zhong Bing Ji Jiu Yi Xue* 2007;**19**:336–9.

17 Li H, Wang S-R, Wang L-K, *et al.* Perihematomal pathological changes in neurons and astrocytes following acute cerebral hemorrhage. *Int J Neurosci* 2010;**120**:683–90. doi:10.3109/00207454.2010.513460

18 Liu B, Hu B, Shao S, *et al.* CD163/Hemoglobin Oxygenase-1 Pathway Regulates Inflammation in Hematoma Surrounding Tissues after Intracerebral Hemorrhage. *J Stroke Cerebrovasc Dis* 2015;**24**:2800–9. doi:10.1016/j.jstrokecerebrovasdis.2015.08.013

19 Liu QX, Zhang HL, Zhang SM. The expressions of hypoxia-inducible factor-1α and its significance in perihematomal issue in human intracerebral hemorrhage. *Chinese Journal of Neurology* 2006;**39**:24–6.

20 Mantle D, Siddique S, Eddeb F, *et al.* Comparison of protein carbonyl and antioxidant levels in brain tissue from intracerebral haemorrhage and control cases. *Clin Chim Acta* 2001;**312**:185–90. doi:10.1016/s0009-8981(01)00623-4

21 McCarron MO, Nicoll JA, Stewart J, *et al.* The apolipoprotein E epsilon2 allele and the pathological features in cerebral amyloid angiopathy-related hemorrhage. *J Neuropathol Exp Neurol* 1999;**58**:711–8. doi:10.1097/00005072-199907000-00005
22 Rosell A, Vilalta A, García-Berrocoso T, *et al.* Brain perihematoma genomic profile following spontaneous human intracerebral hemorrhage. *PLoS One* 2011;**6**:e16750. doi:10.1371/journal.pone.0016750
23 Rosell A, Ortega-Aznar A, Alvarez-Sabín J, *et al.* Increased brain expression of matrix metalloproteinase-9 after ischemic and hemorrhagic human stroke. *Stroke* 2006;**37**:1399–406. doi:10.1161/01.STR.0000223001.06264.af
24 Shen J, Xie L, Mao X, *et al.* Neurogenesis after primary intracerebral hemorrhage in adult human brain. *J Cereb Blood Flow Metab* 2008;**28**:1460–8. doi:10.1038/jcbfm.2008.37
25 Shtaya A, Bridges LR, Esiri MM, *et al.* Rapid neuroinflammatory changes in human acute intracerebral hemorrhage. *Ann Clin Transl Neurol* 2019;**6**:1465–79. doi:10.1002/acn3.50842
26 Tanskanen M, Myllykangas L, Saarialho-Kere U, *et al.* Matrix metalloproteinase-β19 expressed in cerebral amyloid angiopathy. *Amyloid* 2011;**18**:3–9. doi:10.3109/13506129.2010.541960
27 Vakulenko NN. [Pathohistologic and biochemical changes in the brain in atherosclerosis with hemorrhagic foci]. *Zh Nevropatol Psikhiatr Im S S Korsakova* 1977;**77**:993–5.
28 Wang D, Guo F, Sun X, *et al.* Redox factor 1 inhibits the apoptosis process after intracerebral hemorrhage. *Neurol Res* 2011;**33**:681–5. doi:10.1179/1743132810Y.0000000021
29 Wang Y-X, Yan A, Ma Z-H, *et al.* Nuclear factor-κB and apoptosis in patients with intracerebral hemorrhage. *J Clin Neurosci* 2011;**18**:1392–5. doi:10.1016/j.jocn.2010.11.039
30 Wang D, Qi J, Li G. Study on the association of reaction of astrocytes with cyclin D1 protein in peripheral tissue of human brain hemorrhagic area. *Zhonghua Yi Xue Za Zhi* 2004;**84**:1710–2.
31 Wu C, Yan X, Liao Y, *et al.* Increased perihematomal neuron autophagy and plasma thrombin-antithrombin levels in patients with intracerebral hemorrhage: An observational study. *Medicine* 2019;**98**:e17130. doi:10.1097/MD.0000000000017130
32 Wu H, Zhang Z, Hu X, *et al.* Dynamic changes of inflammatory markers in brain after hemorrhagic stroke in humans: a postmortem study. *Brain Res* 2010;**1342**:111–7. doi:10.1016/j.brainres.2010.04.033
33 Wu H, Zhao R, Qi J, *et al.* The expression and the role of protease nexin-1 on brain edema after intracerebral hemorrhage. *J Neurol Sci* 2008;**270**:172–83. doi:10.1016/j.jns.2008.03.010
34 Wu C, Ding X, Wang H, *et al.* [Neural apoptosis and apoptosis-related genes in intracerebral hemorrhage patients]. *Zhonghua Yi Xue Za Zhi* 2006;**86**:3073–6.
35 Yilmaz A, Fuchs T, Dietel B, *et al.* Transient decrease in circulating dendritic cell precursors after acute stroke: potential recruitment into the brain. *Clin Sci* 2009;**118**:147–57. doi:10.1042/CS20090154
36 Zhang XW, Wu Y, Wang DK, *et al.* Expression changes of inflammatory cytokines TNF-α, IL-1β and HO-1 in hematoma surrounding brain areas after intracerebral hemorrhage. *J Biol Regul Homeost Agents* 2019;**33**:1359–67. doi:10.23812/19-150-A
37 Zhang Z-L, Liu Y-G, Huang Q-B, *et al.* Nuclear factor-κB activation in perihematomal brain tissue correlates with outcome in patients with intracerebral hemorrhage. *J Neuroinflammation* 2015;**12**:53. doi:10.1186/s12974-015-0277-9
38 Zhang Z, Liu Y, Huang Q, *et al.* NF-kappaB activation and cell death after intracerebral hemorrhage in patients. *Neurological sciences : official journal of the Italian Neurological Society and of the Italian Society of Clinical Neurophysiology* 2014;**35**:1097–102.
39 Zhang X, Zhang Z, Yin X, *et al.* Exploring the optimal operation time for patients with hypertensive intracerebral hemorrhage: tracking the expression and progress of cell apoptosis of prehematomal brain tissues. *Chin Med J* 2010;**123**:1246–50.
40 Zhang Z, Qi J, Zhu H, *et al.* Expression of thrombin and its associated protein in cerebellum of human and rat after intracerebral hemorrhage. *Chin Med J* 2010;**123**:2077–81.
41 Zhang WW, Huang YH, Li J, *et al.* Altered expression of endothelin-1 in perihematoma astrocytes in patients with intracerebral hemorrhage. *Chinese Journal of Clinical Rehabilitation* 2003;**8**:180–1.
42 Zhang X, Zhang W, Hao X. The expression of glucose transporter protein one in the astrocytes of the human brain with hypertensive intracerebral hemorrhage. *Chinese Journal of Neurology* 2000;**33**:176–8.
43 Zhao J, Mao Q, Qian Z, *et al.* Effect of mild hypothermia on expression of inflammatory factors in surrounding tissue after minimally invasive hematoma evacuation in the treatment of hypertensive intracerebral hemorrhage. *Exp Ther Med* 2018;**15**:4906–10. doi:10.3892/etm.2018.6014
44 Zhu S, Tang Z, Guo S, *et al.* Experimental study on the expression of HIF-1alpha and its

relationship to apoptosis in tissues around cerebral bleeding loci. *J Huazhong Univ Sci Technolog Med Sci* 2004;**24**:373–5. doi:10.1007/BF02861871
